# Efficient and divergent synthesis of polyfunctionalized 2-pyridones from β-keto amides†

**DOI:** 10.1039/c8ra05709e

**Published:** 2018-10-01

**Authors:** Baochang Gao, Yufeng Sun, Jun Wang, Zhigang Yuan, Liwu Zu, Xu Zhang, Wenbin Liu

**Affiliations:** Institute of Composite Materials, Key Laboratory of Superlight Material and Surface Technology of Ministry of Education, College of Materials Science and Chemical Engineering, Harbin Engineering University Harbin 150001 P. R. China chemgbc@hrbeu.edu.cn liuwenbin@hrbeu.edu.cn; Daqing Branch of Heilongjiang Academy of Sciences Daqing 163319 P. R. China

## Abstract

Efficient and divergent one-pot synthesis of polyfunctionalized 2-pyridones from β-keto amides based on reaction condition selection was developed. The methodology offers several significant advantages including mild conditions, ease of handling, high yields, and a relatively broad range of substrates. Based on various experiments and observations, a plausible mechanism for the selective synthesis of 2-pyridones was proposed.

## Abstract 2

Divergent synthesis of 2-pyridones.

## Graphical abstract

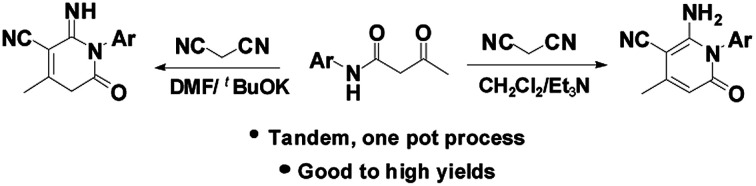

## Introduction

Molecularly diverse and complex heterocycles make a vital contribution to the organic synthesis and discovery of new pharmaceutical reagents.^1^ 2-Pyridone is one of the most significant heteroaromatic rings in natural products, bioactive molecules, and pharmaceutical agents.^2–5^ Such well-known molecules possess a wide spectrum of biological properties such as antimalarial,^6^ anti-hepatitis B,^7^ vasorelaxant,^8^ anti-fungal,^9^ anti-epilepsy,^10^ anti-fibrosis,^11^ anti-HIV,^12^ MEK-1 inhibitor,^13^ antitumor,^14^ anti-ulcer,^15^ antioxidant, and antituberculosis activities.^16^ Moreover, molecules bearing 2-pyridone derivatives are also used as important structural units in the synthesis of nitrogen-containing heterocycles, such as pyridines,^17^ piperidines,^18^ β-lactams,^19^ indolizidines, and quinolizidines.^20^ Thus, the synthesis of 2-pyridone derivatives has attracted considerable attention in recent years. Owing to their biologically and structurally interesting properties, a number of approaches for their construction have been reported. The classical methods for synthesis of 2-pyridones include pyridinium salt chemistry and N-alkylation,^21,22^ Guareschi–Thorpe reaction,^23^ Dieckmann-type condensation,^24^ Diels–Alder reaction,^25^ and halogenated 2-pyridones under Vilsmeier conditions,^26^ and other methods from the synthesis of 2-pyridones under metal catalyst.^27^ Each of these approaches represents an important progress for the synthesis of 2-pyridones, however, the development of an efficient and facile strategies are desirable for synthesis of the valuable heterocycles. The development of an efficient and facile methodology for the construction of diverse 2-pyridone derivatives still remains a great challenge.

β-Keto amide and their derivatives have been proven to be the important starting materials and reagents in the construction of heterocyclic systems since they possess six reactive sites in the same molecule.^28–35^ They have also been used as building blocks for the construction of biologically interesting heterocycles.^36^ So far, a number of natural product-like heterocyclic compounds have been successfully synthesized based on β-keto amide and their derivatives. Their synthetic methodology and associated medicinal activity should be engaged in the chemistry community.^37^ As part of our efforts to discover novel and practical synthesis methods for the construction of heterocyclic compounds, we recently developed an efficient synthesis of substituted thieno[2,3-*b*]pyridines from β-keto amides.^38^ We are interested in the fields of cleavage or construction of C–C and C–N bonds because of its varied applications.^39^ The development of a catalytic process for simultaneous cleavage and building of C–N and C–C bonds is a significant challenge in synthetic chemistry.^40^ Liang *et al.* reported a new series of compounds *via* three-component one-pot reactions of 1-acetyl-1-carbamoyl cyclopropanes, malononitrile and cyclic secondary amines.^41^ Given that the approach of Liang involved the tandem reaction of β-keto amide reactants to the corresponding fully substituted 2-pyridones, we supposed that the direct reaction of β-keto amides with malononitrile through control reaction conditions would afford 2-pyridones with diverse structures through the control of reaction conditions. When piperidine was replaced with other bases, the polysubstituted 2-pyridones with different substituted patterns could be obtained by using β-keto amides as raw material.

**Scheme 1:**
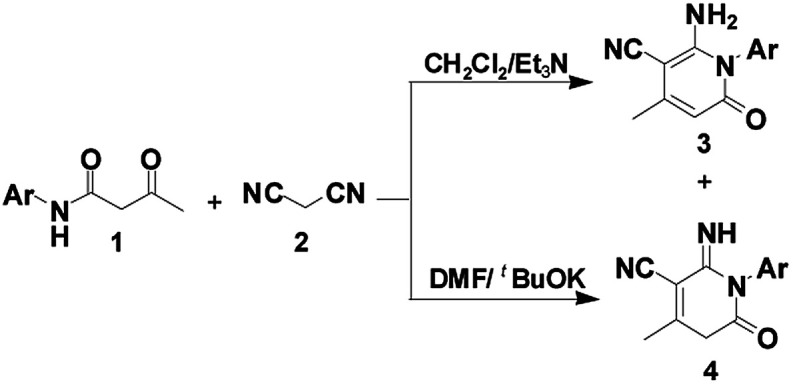
Proposed mechanism for the construction of 2-pyridones 3 and 4.

Inspired by these findings, we envisioned the construction of the 2-pyridone core from β-keto amides through intramolecular nucleophilic cyclization. As the result of our continued interest in this area, we have provided an efficient and selective synthesis methodology for the construction of two libraries of 2-pyridones 3 and 4*via* a tandem annulation of β-keto amides 1 and malononitrile 2 in CH_2_Cl_2_ and DMF, respectively (Scheme 1). Herein, we wish to report our results and the possible mechanism involved was discussed.

## Results and discussion

To reduce this idea to practice, we examined the reactivity of the β-keto amides 1 and 2. We then selected 3-oxo-*N*-phenyl-butyramide 1a as the model substrate to screen its reaction conditions. The reaction was executed with EtONa in anhydrous ethanol at room temperature. Unfortunately the reactions was unsuccessful, no reaction occurred even till 2 h as indicated by TLC (Table 1, entry 1). When 1a was heated with 2 (1.1 equiv.) in EtOH at reflux for 6 h, we were delighted to have the desired 2-pyridone 3a, although the yield was very low (38%, Table 1, entry 2). Surprisingly the transformation was observed using EtONa to generate the reaction. The product was characterized as 2-amino-4-methyl-6-oxo-1-phenyl-1,6-dihydro-pyridine-3-carbonitrile 3a on the basis of its NMR spectra and analytical data (Table 1, entry 2).

**Table 1:** Optimization of the reaction conditions for the model reaction^a^

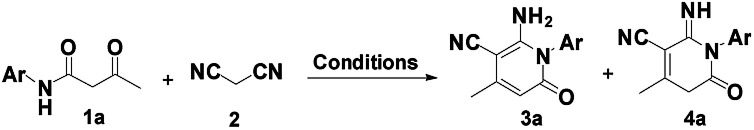					
Entry	Solvent	Mediated^b^	Temp. (°C)	Yield^c^ (%)	
				3a	4a
1	EtOH	EtONa	rt	n.d.	n.d.
2	EtOH	EtONa	Reflux	38	n.d.
3	EtOH	DBU	Reflux	50	n.d.
4	EtOH	Et_3_N	Reflux	55	n.d.
5	CH_2_Cl_2_	Cs_2_CO_3_	Reflux	63	n.d.
6	CH_2_Cl_2_	DBU	Reflux	72	n.d.
7	CH_2_Cl_2_	Et_3_N	Reflux	88	n.d.
8	DMF	Piperidine	rt	32	10
9	DMF	Cs_2_CO_3_	70	45	n.d.
10	DMF	K_2_CO_3_	70	41	n.d.
11	DMF	^*t*^BuOK	rt	n.d.	85
12	DMF	^*t*^BuOK	70	n.d.	87

a The reaction was performed with 1a (1.0 mmol), 2 (1.1 mmol), and the solvent (10 mL).

b Mediated (1.0 mmol) was added to the reaction.

c Isolated yields were based on β-keto amide 1a.

To optimize the reaction conditions, the reaction of β-keto amide derivative 1a with 2 was investigated using various bases and solvent (Table 1). Initially, different organic bases, such as DBU and Et_3_N, were employed as in EtOH. When EtONa was replaced by DBU or Et_3_N, the conversion of (2-amino-4-methyl-6-oxo-1-phenyl-1,6-dihydro-pyridine-3-carbonitrile) 3a was increased, affording compound 3a in yields of 50% and 55%, respectively (Table 1, entries 3–4). The reaction conditions were then optimized by screening several solvents. Hence, treatment of 1a with 2 in the presence of Cs_2_CO_3_ or DBU in CH_2_Cl_2_ at reflux, product 3a was formed in 63 and 72% yield, respectively (Table 1, entries 5–6). The best yield was achieved with Et_3_N in refluxing CH_2_Cl_2_, the yield of desired product 3a reached 88% yield (Table 1, entry 7). When the reaction was performed in the presence of piperidine in DMF at room temperature, a new compound 4a was successfully isolated in 10% yield (Table 1, entry 8). In order to efficiently obtain a single final product 4a, additional attempts were made. The introduction of carbonate such as Cs_2_CO_3_ or K_2_CO_3_ did not improve the selectivity and the conversion (Table 1, entries 9–10). However, product 4a was obtained in 85% yield by using ^*t*^BuOK in DMF at room temperature (Table 1, entry 11). Thus, dichloromethane was the best medium for selectively obtaining product 3a. Conversely, the best conditions for the preparation of 4a were conducted in DMF at room temperature.

Under the optimal reaction conditions (Table 1, entry 7), the 2-pyridone 3 was tested by using a range of readily available β-keto amides and malononitrile. In the following work, the scope of the substrate was investigated (Table 2). Under the conditions, a series of reactions of β-keto amides with different substitution groups on the phenyl ring were examined, and the results are listed in Table 2. As shown in Table 2, it was observed that the substrate with various electron-donating and -withdrawing groups 1b–1h could easily be converted into the corresponding 2-pyridones 3b–3h in high yields (85–95%) in 6–7.5 hours (Table 2, entries 2–8). Furthermore, the positions of the substituents on the phenyl ring did not show any effects on the reaction. It is noteworthy that all the crude products 3 could be purified simply by recrystallization from ethanol.

**Table 2:** Substrate scope of 1 and synthesis of target molecules 3^a^

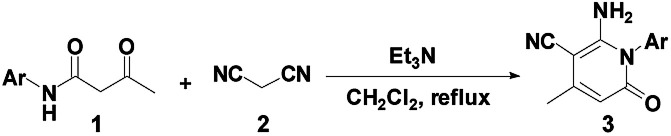					
Entry	1	Ar	3	Temp. (°C)	Yield^b^ (%)
1	1a	Ph	3a	Reflux	88
2	1b	4-ClC_6_H_4_	3b	Reflux	95
3	1c	4-MeOC_6_H_4_	3c	Reflux	85
4	1d	2-ClC_6_H_4_	3d	Reflux	88
5	1e	2,4-Me_2_C_6_H_3_	3e	Reflux	91
6	1f	4-MeC_6_H_4_	3f	Reflux	85
7	1g	2-MeOC_6_H_4_	3g	Reflux	87
8	1h	2-MeC_6_H_4_	4h	Reflux	85

a All reactions were carried out with 1 (1.0 mmol), 2 (1.1 mmol), Et_3_N (1.0 equiv.), in CH_2_Cl_2_ (5.0 mL) at reflux for 6 hours.

b Isolated yield.

Next, with the aim to make product 4 to be the main product (Scheme 1), the selective synthesis of 2-pyridone 4a under appropriate conditions may be realized. Basic conditions play an important role in the transformation process. The use of Et_3_N selectively afforded compound 3 in high conversion. After several trials, we found that when Et_3_N was replaced by a stronger base such as ^*t*^BuOK, product 4a could be obtained in a yield of 85% (Table 1, entry 11). Effect of temperature showed no improvement in the yield of product 4a at room temperature and 70 °C (Table 1, entry 12). Under the optimal reaction conditions, a series of reactions of β-keto amides with different substitution groups on the phenyl ring were examined, affording 2-pyridones 4b–4h in good-to-high yields (85–95%). Notably, all the crude products 4 could be purified simply by recrystallization from DMF (Table 3).

**Table 3:** Substrate scope of 1 and synthesis of target molecules 4^a^

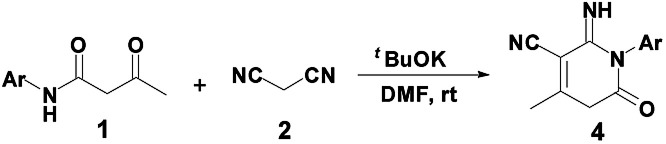					
Entry	1	Ar	4	Temp. (°C)	Yield^b^ (%)
1	1a	Ph	4a	rt	88
2	1b	4-ClC_6_H_4_	4b	rt	95
3	1c	4-MeOC_6_H_4_	4c	rt	85
4	1d	2-ClC_6_H_4_	4d	rt	88
5	1e	2,4-Me_2_C_6_H_3_	4e	rt	91
6	1f	4-MeC_6_H_4_	4f	rt	85
7	1g	2-MeOC_6_H_4_	4g	rt	87
8	1h	2-MeC_6_H_4_	4h	rt	85

a All reactions were carried out with 1 (1.0 mmol), 2 (1.1 mmol), ^*t*^BuOK (1.0 equiv.), in CH_2_Cl_2_ (5.0 mL) at room temperature.

b Isolated yield.

**Scheme 2:**
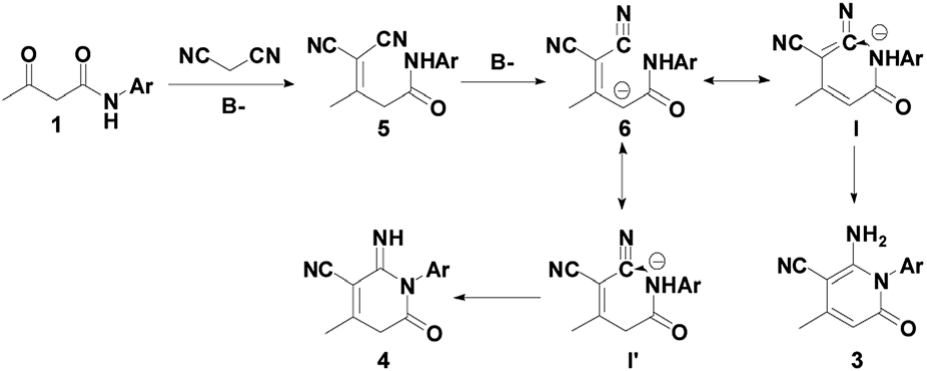
Plausible mechanism for the construction of 2-pyridones 3 and 4.

On the basis of the obtained results and previously reported work,^41,42^ a plausible mechanism for the syntheses of 2-pyridones 3 and 4 are presented, as depicted in Scheme 2. First, the β-keto amide 1 reacts with malononitrile 2 accompanying the loss of one molecule of H_2_O to generate intermediate 5, and affords intermediate 6. Then, the isomerization of I in the presence of Et_3_N forms final product 3 under the control experiments conditions, which is cyclized by intramolecular nucleophilic process. Likewise, the isomerization of I′ in the presence of the ^*t*^BuOK affords final product 4.

## Conclusions

A simple and efficient one-pot methodology for the divergent synthesis of polysubstituted 2-pyridones 3 and 4 in high yield has been developed. This method illustrates a practical protocol using simple and inexpensive starting materials. Furthermore, the methodology offers several significant advantages, such as economic availability, metal catalyst-free, ease of handling, a relative broad range of substrates, and environmental benignity under mild reaction condition. Further expanding the scope of this reaction and application the products are currently on-going in our laboratory.

## Experimental

### General

All reagents were purchased from commercial sources and used without treatment, unless otherwise indicated. The products were purified by recrystallization. ^1^H and ^13^C NMR spectra were recorded on a Bruker AVANCE-300 spectrometer at 25 °C using TMS as an internal standard and deuterated DMSO (DMSO-*d*_6_) as the solvent. Petroleum ether (PE) used was the fraction boiling in the range 30–60 °C. Elemental analyses were obtained on a Vario EL analyzer.

### General procedure for the synthesis of compounds 3a–h

To a solution of β-keto amides 1 (1.0 mmol) and malononitrile (1.1 mmol) in CH_2_Cl_2_ (10 mL) was added Et_3_N (1.0 mmol) in one portion. The mixture was well stirred for 6 h at reflux. When consumption of 1 was complete (TLC), the mixture was washed with saturated sodium chloride solution (10 mL × 3) and dried over anhydrous MgSO_4_. The organic solvent CH_2_Cl_2_ was then removed under reduced pressure and the residue was purified by recrystallization from ethanol to give product 3a–h.

#### 2-Amino-4-methyl-6-oxo-1-phenyl-1,6-dihydro-pyridine-3-carbonitrile (3a)

White solid; mp 281–282 °C; ^1^H NMR (300 MHz, DMSO-*d*_6_): *δ* = 7.57–7.47 (m, 3H), 7.24 (t, *J* = 7.0 Hz, 2H), 6.68 (s, 2H), 5.67 (s, 1H), 2.17 (s, 3H); ^13^C NMR (75 MHz, DMSO-*d*_6_): *δ* = 161.1, 156.3, 152.1, 135.2, 130.6, 129.9, 129.3, 129.1, 118.0, 117.7, 105.5, 71.8, 20.9; anal. calcd for C_13_H_11_N_3_O: C, 76.98; H, 7.00; N, 7.48; found: C, 76.96; H, 7.01; N, 7.49.

#### 2-Amino-4-methyl-6-oxo-1-phenyl-1,6-dihydro-pyridine-3-carbonitrile (3b)

White solid; mp 328–329 °C; ^1^H NMR (300 MHz, DMSO-*d*_6_): *δ* = 7.62–7.57 (m, 2H), 7.31–7.26 (m, 2H), 6.91 (s, 2H), 5.66 (d, *J* = 1.1 Hz, 1H), 2.18 (s, 3H); ^13^C NMR (75 MHz, DMSO-*d*_6_): *δ* = 161.0, 156.4, 152.4, 134.5, 134.3, 131.4, 130.6, 117.6, 105.2, 71.8, 20.9; anal. calcd for C_13_H_10_ClN_3_O: C, 60.12; H, 3.88; N, 16.18; found: C, 60.11; H, 3.90; N, 16.17; MS: *m*/*z* calcd for C_13_H_10_ClN_3_O, 259.05; found, 260.1 [M + 1].

#### 2-Amino-1-(4-methoxy-phenyl)-4-methyl-6-oxo-1,6-dihydro-pyridine-3-carbonitrile (3c)

White solid; mp 273–274 °C; ^1^H NMR (300 MHz, DMSO-*d*_6_): *δ* = 7.16–7.12 (m, 2H), 7.09–7.05 (m, 2H), 6.68 (s, 2H), 5.65–5.64 (d, *J* = 3 Hz, 1H), 3.81 (s, 3H), 2.16 (s, 3H); ^13^C NMR (75 MHz, DMSO-*d*_6_): *δ* = 161.3, 160.1, 156.7, 152.0, 130.4, 127.5, 117.7, 115.8, 105.4, 71.6, 55.8, 20.9; anal. calcd for C_14_H_13_N_3_O_2_: C, 65.87; H, 5.13; N, 16.46; found: C, 65.88; H, 5.11; N, 16.47.

#### 3- Amino-1-(2-chloro-phenyl)-4-methyl-6-oxo-1,6-dihydro-pyridine-3-carbonitrile (3d)

White solid; mp 265–266 °C; ^1^H NMR (300 MHz, DMSO-*d*_6_): *δ* = 7.65 (dd, *J*_1_= 7.3 Hz, *J*_2_= 1.9 Hz, 1H), 7.54–7.48 (m, 2H), 7.40 (dd, *J*_1_ = 7.2 Hz, *J*_2_ = 2.2 Hz, 1H), 7.00 (s, 2H), 5.66 (s, 1H), 2.18 (s, 3H); ^13^C NMR (75 MHz, DMSO-*d*_6_): *δ* = 160.3, 156.1, 152.8, 132.9, 132.5, 131.8, 131.6, 131.1, 129.5, 117.6, 105.1, 71.6, 21.0; anal. calcd for C_13_H_10_ClN_3_O: C, 60.12; H, 3.88; N, 16.18; found: C, 60.11; H, 3.90; N, 16.17.

#### 2-Amino-1-(2,4-dimethyl-phenyl)-4-methyl-6-oxo-1,6-dihydro-pyridine-3-carbonitrile (3e)

White solid; mp 296–297 °C; ^1^H NMR (400 MHz, DMSO-*d*_6_): *δ* = 7.21 (s, 1H), 7.14 (d, *J* = 7.9 Hz, 1H), 6.99 (d, *J* = 8.0 Hz, 1H), 6.67 (s, 2H), 5.65 (s, 1H), 2.16 (s, 3H), 1.91 (s, 3H); ^13^C NMR (100 MHz, DMSO-*d*_6_): *δ* = 160.6, 156.1, 152.2, 139.4, 135.8, 132.5, 131.6, 128.9, 128.8, 117.7, 105.4, 71.6, 21.2, 20.9, 17.1; anal. calcd for C_15_H_15_N_3_O: C, 71.13; H, 5.97; N, 16.59; found: C, 71.15; H, 5.96; N, 16.60.

#### 2-Amino-4-methyl-6-oxo-1-*p*-tolyl-1,6-dihydro-pyridine-3-carbonitrile (3f)

White solid; mp 264–265 °C; ^1^H NMR (300 MHz, DMSO-*d*_6_): *δ* = 7.35 (d, *J* = 8.0 Hz, 2H), 7.13–7.09 (m, 2H), 6.66 (s, 2H), 5.66 (d, *J* = 1.1 Hz, 1H), 2.38 (s, 3H), 2.16 (d, *J* = 0.9 Hz, 3H); ^13^C NMR (75 MHz, DMSO-*d*_6_): *δ* = 161.1, 156.4, 152.0, 139.2, 132.5, 131.1, 128.9, 117.7, 105.5, 71.7, 21.3, 20.9. anal. calcd for C_14_H_13_N_3_O: C, 70.28; H, 5.48; N, 17.56; found: C, 70.26; H, 5.49; N, 17.57.

#### 2-Amino-1-(2-methoxy-phenyl)-4-methyl-6-oxo-1,6-dihydro-pyridine-3-carbonitrile (3g)

White solid; mp 281–282 °C; ^1^H NMR (300 MHz, DMSO-*d*_6_): *δ* = 7.52–7.47 (m, 1H), 7.24–7.19 (m, 1H), 7.18–7.16 (m, 1H), 7.12–7.06 (m, 1H), 6.72 (s, 2H), 5.64 (s, 1H), 3.75 (s, 3H), 2.18 (s, 3H); ^13^C NMR (75 MHz, DMSO-*d*_6_): *δ* = 160.7, 156.4, 155.4, 152.1, 131.5, 130.5, 123.2, 121.8, 117.8, 113.6, 105.4, 71.5, 56.2, 20.9; anal. calcd for C_14_H_13_N_3_O_2_: C, 65.87; H, 5.13; N, 16.46; found: C, 65.88; H, 5.11; N, 16.47.

#### 2-Amino-4-methyl-6-oxo-1-*o*-tolyl-1,6-dihydro-pyridine-3-carbonitrile (3h)

White solid; mp 284–285 °C; ^1^H NMR (300 MHz, DMSO-*d*_6_): *δ* = 7.43 (d, *J* = 4.6 Hz, 2H), 7.40–7.38 (m, 1H), 7.16 (d, *J* = 7.4 Hz, 1H), 6.72 (s, 2H), 5.71 (s, 1H), 2.21 (s, 3H), 2.00 (s, 3H); ^13^C NMR (75 MHz, DMSO-*d*_6_): *δ* = 160.5, 156.0, 152.4, 136.2, 134.2, 131.9, 130.1, 129.2, 128.2, 117.6, 105.5, 71.7, 20.9, 17.2; anal. calcd for C_14_H_13_N_3_O: C, 70.28; H, 5.48; N, 17.56; found: C, 70.26; H, 5.49; N, 17.57.

### General procedure for the synthesis of compounds 4a–h

To a solution of β-keto amides 1 (1.0 mmol) and malononitrile (1.1 mmol) in DMF (10 mL) was added ^*t*^BuOK (1.0 mmol) in one portion. The mixture was well stirred for 4 hours at room temperature. When consumption of 1 was complete (TLC), the mixture was poured into sat. aq. NaCl (50 mL) under stirring. The precipitated solid was collected by filtration, washed with water (3 × 20 mL) and dried *in vacuo* to afford the product 4a–h.

#### 2-Imino-4-methyl-6-oxo-1-phenyl-1,2,5,6-tetrahydro-pyridine-3-carbonitrile (4a)

White solid; mp 130–131 °C; ^1^H NMR (300 MHz, DMSO-*d*_6_): *δ* = 10.36 (s, 1H), 7.57 (d, *J* = 7.8 Hz, 2H), 7.33 (t, *J* = 7.8 Hz, 2H), 7.09 (t, *J* = 7.3 Hz, 1H), 3.77 (s, 2H), 2.36 (s, 3H); ^13^C NMR (75 MHz, DMSO-*d*_6_): *δ* = 178.3, 164.7, 138.9, 129.3, 124.3, 119.8, 112.6, 112.6, 87.2, 45.1, 24.1; anal. calcd for C_13_H_11_N_3_O: C, 69.32; H, 4.92; N, 18.66; found: C, 69.31; H, 4.94; N, 18.65; MS: *m*/*z* calcd for C_13_H_11_N_3_O, 225.09; found, 226.0 [M + 1].

#### 1-(4-Chloro-phenyl)-2-imino-4-methyl-6-oxo-1,2,5,6-tetrahydro-pyridine-3-carbonitrile (4b)

White solid; mp 125–126 °C; ^1^H NMR (300 MHz, DMSO-*d*_6_): *δ* = 10.50 (s, 1H), 7.60 (d, *J* = 8.8 Hz, 2H), 7.39 (d, *J* = 8.8 Hz, 2H), 3.78 (s, 2H), 2.36 (s, 3H); ^13^C NMR (75 MHz, DMSO-*d*_6_): *δ* = 178.1, 164.9, 137.9, 129.2, 127.9, 121.4, 112.6, 112.5, 87.3, 45.0, 24.1; anal. calcd for C_13_H_10_ClN_3_O: C, 60.12; H, 3.88; N, 16.18; found: C, 60.13; H, 3.88; N, 16.17.

#### 2-Imino-1-(4-methoxy-phenyl)-4-methyl-6-oxo-1,2,5,6-tetrahydro-pyridine-3-carbonitrile (4c)

White solid; mp 138–139 °C; ^1^H NMR (300 MHz, DMSO-*d*_6_): *δ* = 10.21 (s, 1H), 7.48 (d, *J* = 9.0 Hz, 2H), 6.90 (d, *J* = 9.0 Hz, 2H), 3.73 (s, 2H), 3.72 (s, 3H), 2.35 (s, 3H); ^13^C NMR (75 MHz, DMSO-*d*_6_): *δ* = 178.4, 164.2, 156.0, 132.0, 121.4, 114.4, 112.6, 112.6, 87.0, 55.6, 45.0, 24.1; anal. calcd for C_14_H_13_N_3_O_2_: C, 65.87; H, 5.13; N, 16.46; found: C, 65.86; H, 5.15; N, 16.45.

#### 1-(2-Chloro-phenyl)-2-imino-4-methyl-6-oxo-1,2,5,6-tetrahydro-pyridine-3-carbonitrile (4d)

White solid; mp 145–146 °C; ^1^H NMR (300 MHz, DMSO-*d*_6_): *δ* = 10.04 (s, 1H), 7.65 (dd, *J*_1_ = 8.0, *J*_2_ = 1.7 Hz, 1H), 7.51 (dd, *J*_1_ = 7.9, *J*_2_ = 1.5 Hz, 1H), 7.36–7.28 (m, 1H), 7.27–7.19 (m, 1H), 3.83 (s, 2H), 2.36 (s, 3H); ^13^C NMR (75 MHz, DMSO-*d*_6_): *δ* = 178.0, 165.3, 134.7, 130.0, 128.0, 127.6, 127.5, 127.2, 112.6, 112.5, 87.2, 44.6, 24.0; anal. calcd for C_13_H_10_ClN_3_O: C, 60.12; H, 3.88; N, 16.18; found: C, 60.13; H, 3.88; N, 16.17.

#### 1-(2,4-Dimethyl-phenyl)-2-imino-4-methyl-6-oxo-1,2,5,6-tetrahydro-pyridine-3-carbonitrile (4e)

White solid; mp 137–138 °C; ^1^H NMR (300 MHz, DMSO-*d*_6_): *δ* = 9.67 (s, 1H), 7.22 (d, *J* = 8.0 Hz, 1H), 7.04 (s, 1H), 6.97 (d, *J* = 8.1 Hz, 1H), 3.77 (s, 2H), 2.37 (s, 3H), 2.25 (s, 3H), 2.16 (s, 3H); ^13^C NMR (75 MHz, DMSO-*d*_6_): *δ* = 178.6, 164.8, 135.4, 133.5, 132.5, 131.4, 127.0, 125.8, 112.7, 112.6, 87.0, 44.6, 24.1, 21.0, 18.2; anal. calcd for C_15_H_15_N_3_O: C, 71.13; H, 5.97; N, 16.59; found: C, 71.12; H, 5.99; N, 16.58.

#### 2-Imino-4-methyl-6-oxo-1-*p*-tolyl-1,2,5,6-tetrahydro-pyridine-3-carbonitrile (4f)

White solid; mp 100–101 °C; ^1^H NMR (300 MHz, DMSO-*d*_6_): *δ* = 10.25 (s, 1H), 7.45–7.41 (m, 2H), 7.13–7.10 (m, 2H), 3.73 (s, 2H), 2.34 (s, 3H), 2.24 (s, 3H); ^13^C NMR (75 MHz, DMSO-*d*_6_): *δ* = 178.4, 164.5, 136.4, 133.3, 129.7, 119.8, 112.6, 112.6, 87.1, 45.1, 24.1, 20.9; anal. calcd for C_14_H_13_N_3_O: C, 70.28; H, 5.48; N, 17.56; found: C, 70.30; H, 5.47; N,17.55.

#### 2-Imino-1-(2-methoxy-phenyl)-4-methyl-6-oxo-1,2,5,6-tetrahydro-pyridine-3-carbonitrile (4g)

White solid; mp 101–102 °C; ^1^H NMR (300 MHz, DMSO-*d*_6_): *δ* = 9.70 (s, 1H), 7.89 (d, *J* = 7.2 Hz, 1H), 7.16–7.06 (m, 2H), 6.96–6.91 (m, 1H), 3.88 (s, 2H), 3.86 (s, 3H), 2.36 (s, 3H); ^13^C NMR (75 MHz, DMSO-*d*_6_): *δ* = 178.5, 165.1, 150.5, 127.0, 125.7, 123.0, 120.7, 112.7, 112.6, 111.8, 87.1, 56.2, 45.0, 23.9; anal. calcd for C_14_H_13_N_3_O_2_: C, 65.87; H, 5.13; N, 16.46; found: C, 65.86; H, 5.15; N, 16.45.

#### 2-Imino-4-methyl-6-oxo-1-*o*-tolyl-1,2,5,6-tetrahydro-pyridine-3-carbonitrile (4h)

White solid; mp 154–155 °C;^1^H NMR (300 MHz, DMSO-*d*_6_): *δ* = 9.75 (s, 1H), 7.37 (d, *J* = 7.4 Hz, 1H), 7.23 (d, *J* = 7.2 Hz, 1H), 7.13 (dd, *J*_1_ = 12 Hz, *J*_2_ = 6 Hz, 2H), 3.80 (s, 2H), 2.37 (s, 3H), 2.22 (s, 3H); ^13^C NMR (75 MHz, DMSO-*d*_6_): *δ* = 178.5, 164.9, 136.1, 132.5, 130.9, 126.5, 126.2, 125.7, 112.7, 112.6, 87.0, 44.6, 24.1, 18.3; anal. calcd for C_14_H_13_N_3_O: C, 70.28; H, 5.48; N, 17.56; found: C, 70.30; H, 5.47; N, 17.55.

## Conflicts of interest

There are no conflicts to declare.

## Supplementary Material

RA-008-C8RA05709E-s001
