# Supplementary material for: Efficient and divergent synthesis of polyfunctionalized 2-pyridones from β-keto amides
Source: RSC Adv. 2018 Oct 1;8(59):33625–30. doi: 10.1039/c8ra05709e (PMC9086556; doi:10.1039/c8ra05709e)
Supplement: RA-008-C8RA05709E-s001 [file RA-008-C8RA05709E-s001.pdf]

Electronic Supplementary Material (ESI) for RSC Advances.
This journal is © The Royal Society of Chemistry 2018

Efficient and divergent synthesis of polyfunctionalized 2-pyridones from β-keto Amides

Baochang Gao*[a,b], Yufeng Sun[b], Jun Wang[a], Zhigang Yuan[b], Liwu Zu[a], Xu Zhang[b], Wenbin Liu*[a]

[a] Institute of Composite Materials, Key Laboratory of Superlight Material and Surface Technology of Ministry of Education, College of Materials Science and Chemical Engineering, Harbin Engineering University, Harbin 150001, P. R. of China

[b] Daqing Branch of Heilongjiang Academy of Sciences, Daqing 163319, P. R. of China

Email Address: chemgbc@hrbeu.edu.cn; liuwenbin@hrbeu.edu.cn

Supporting Information

Table of contents

I. Experimental section.......................S2

II. ^{1}H NMR and ^{13}C NMR spectra copies of synthesized compounds.......................S3-S18

I. Experimental section

1. General procedure for the preparation of 3a-h

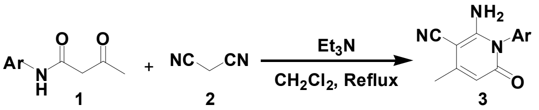

To a solution of β-Keto Amides **1** (1.0 mmol) and malononitrile **2** (1.1 mmol) in CH_2Cl_2 (10 mL) was added Et_3N (1.0 mmol) in one portion. The mixture was well stirred for 6 hours at reflux. When consumption of 1 was complete (TLC), the mixture was washed with saturated sodium chloride solution (10 mL × 3) and dried over anhydrous $MgSO_4$. The organic solvent CH_2Cl_2 was then removed under reduced pressure and the residue was purified by recrystallization from ethanol to give product **3a-h**.

2. General procedure for the preparation of 4a-h

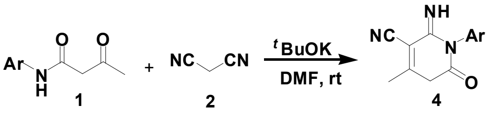

To a solution of β-Keto Amides **1** (1.0 mmol) and malononitrile **2** (1.1 mmol) in DMF (10 mL) was added ^{t}BuOK (1.0 mmol) in one portion. The mixture was well stirred for 4 hours at room temperature. When consumption of 1 was complete (TLC), the mixture was poured into sat. aq NaCl (50 mL) under stirring. The precipitated solid was collected by filtration, washed with water (3×20 mL) and dried in vacuo to afford the product **4a-h**.

II. ^{1}H NMR and ^{13}C NMR spectra copies

Compound **3a**

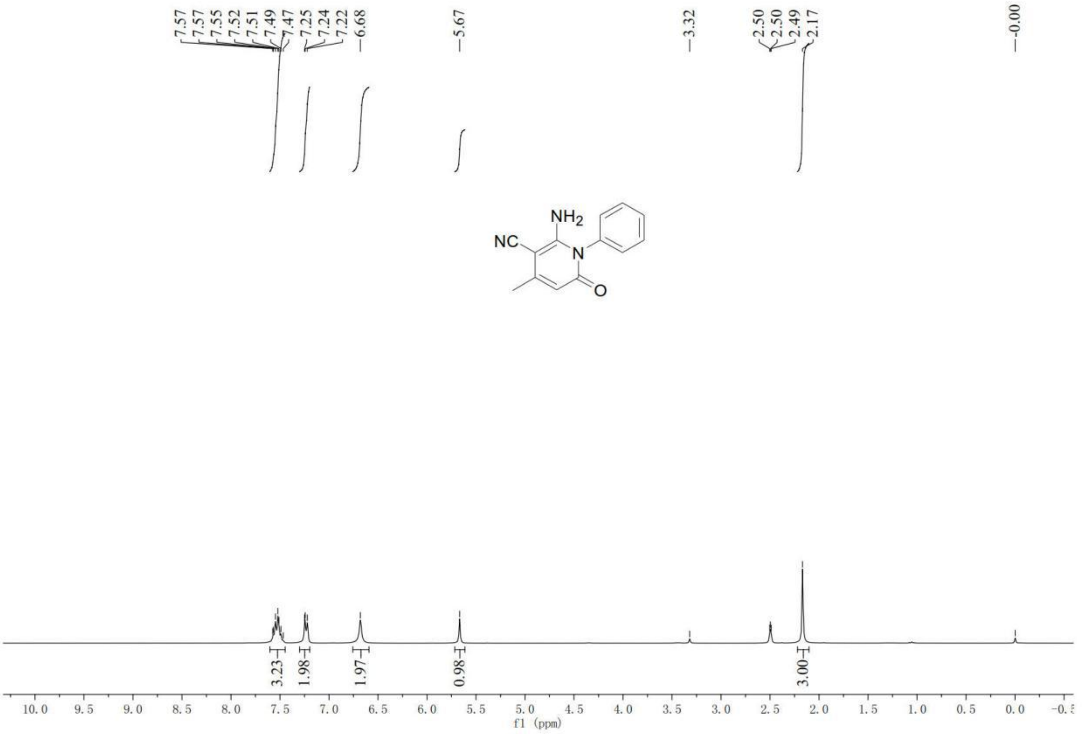

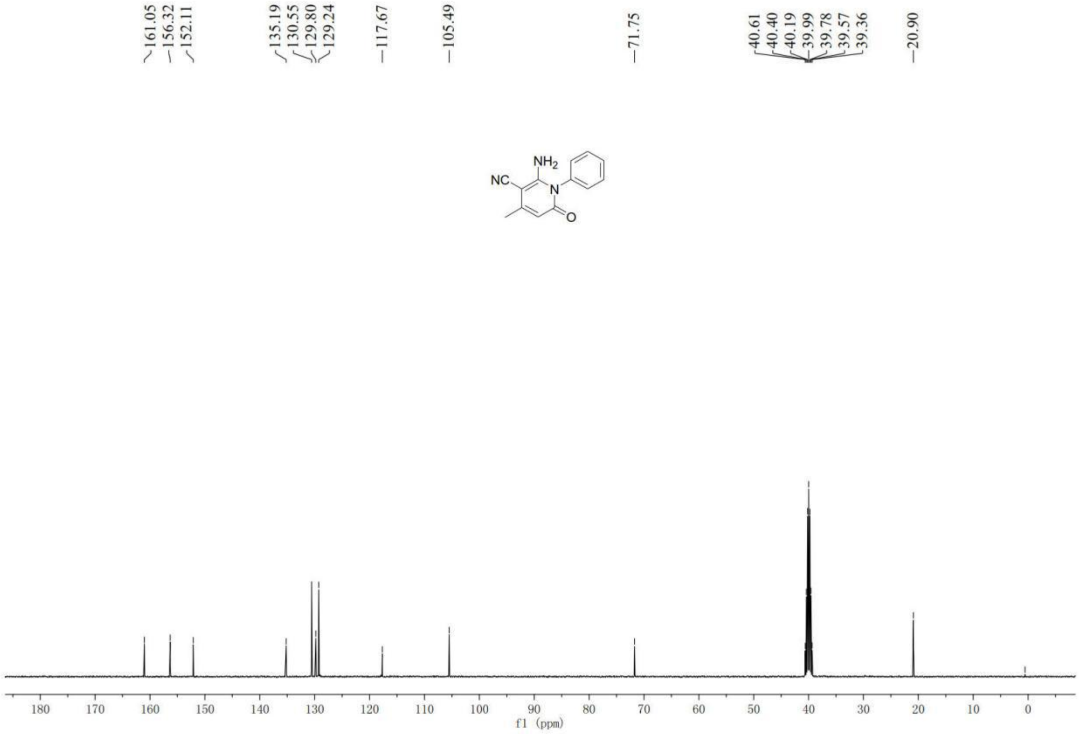

Compound **3b**

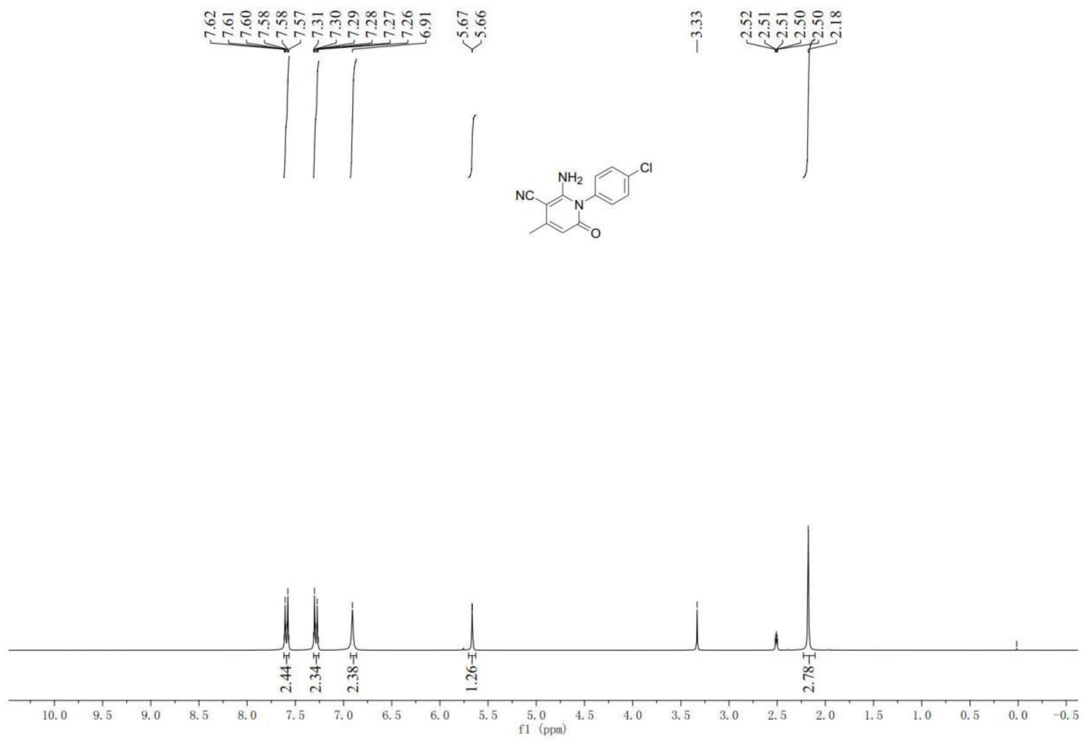

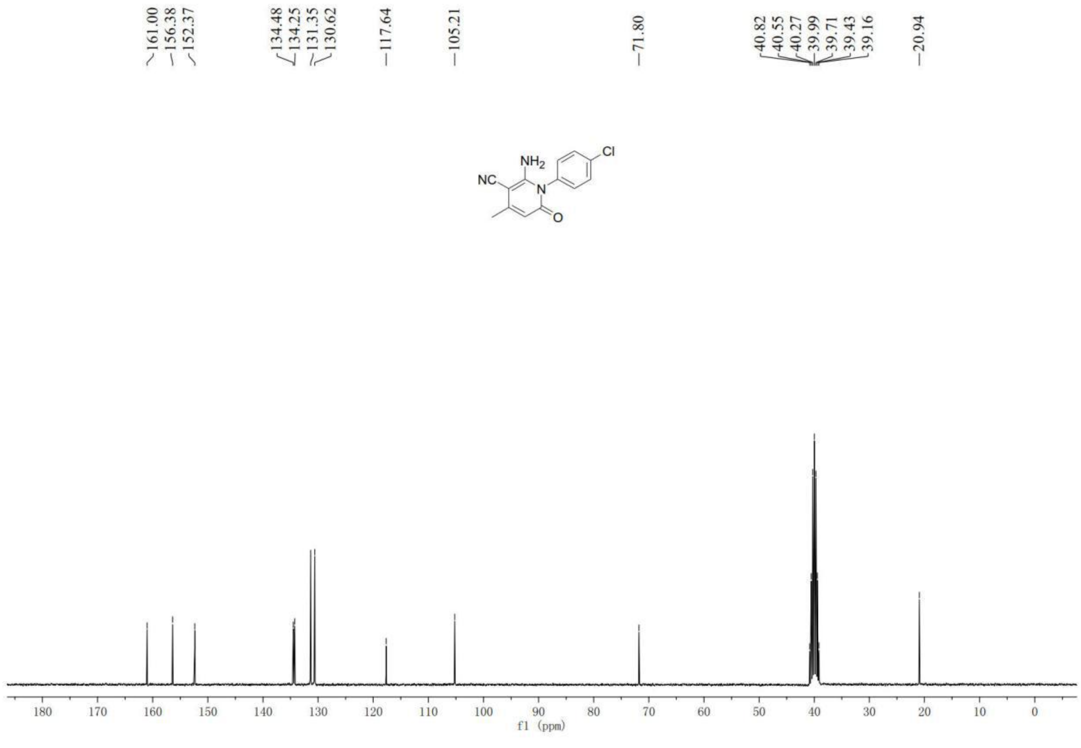

Compound **3c**

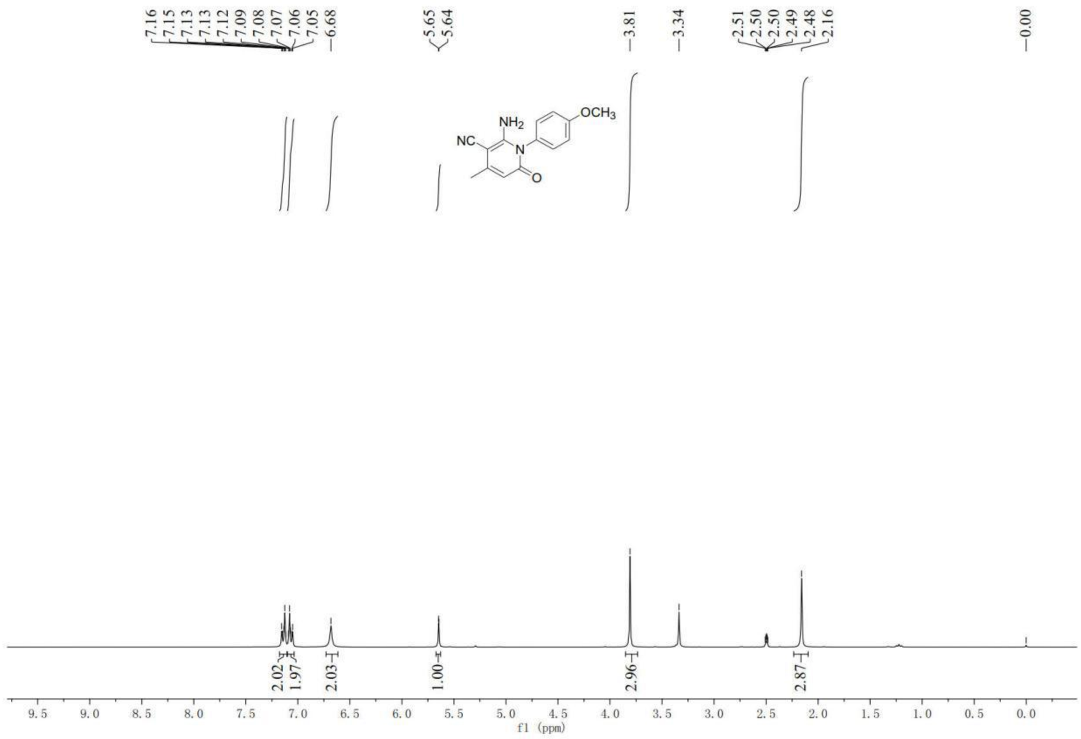

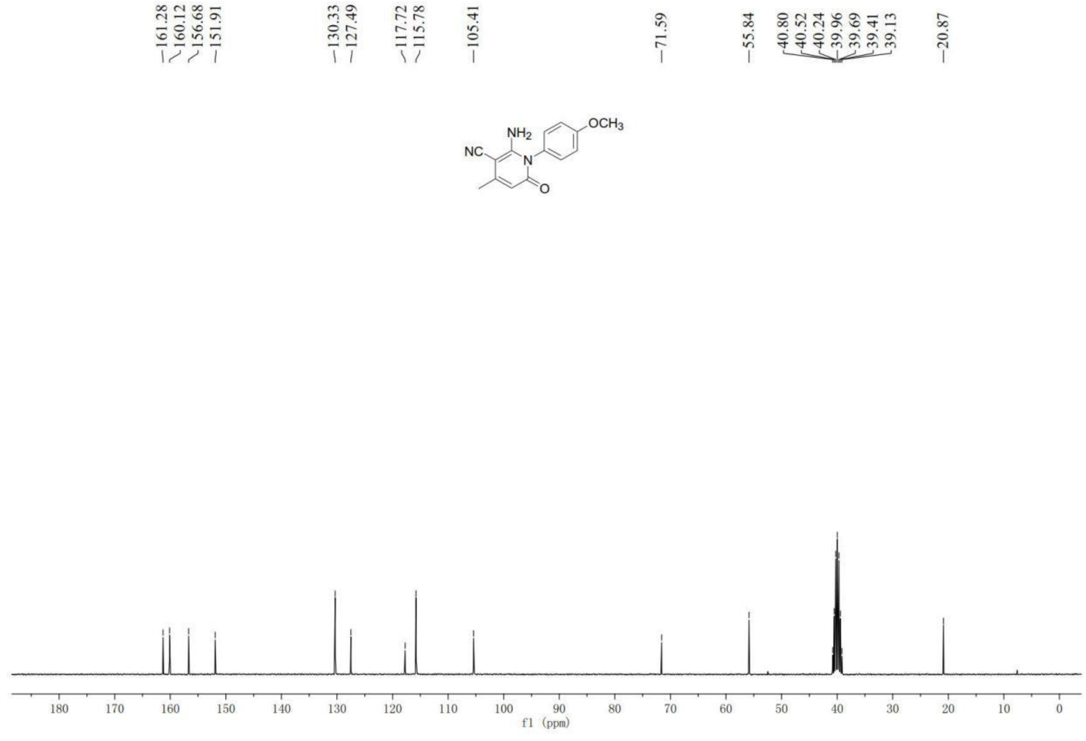

Compound **3d**

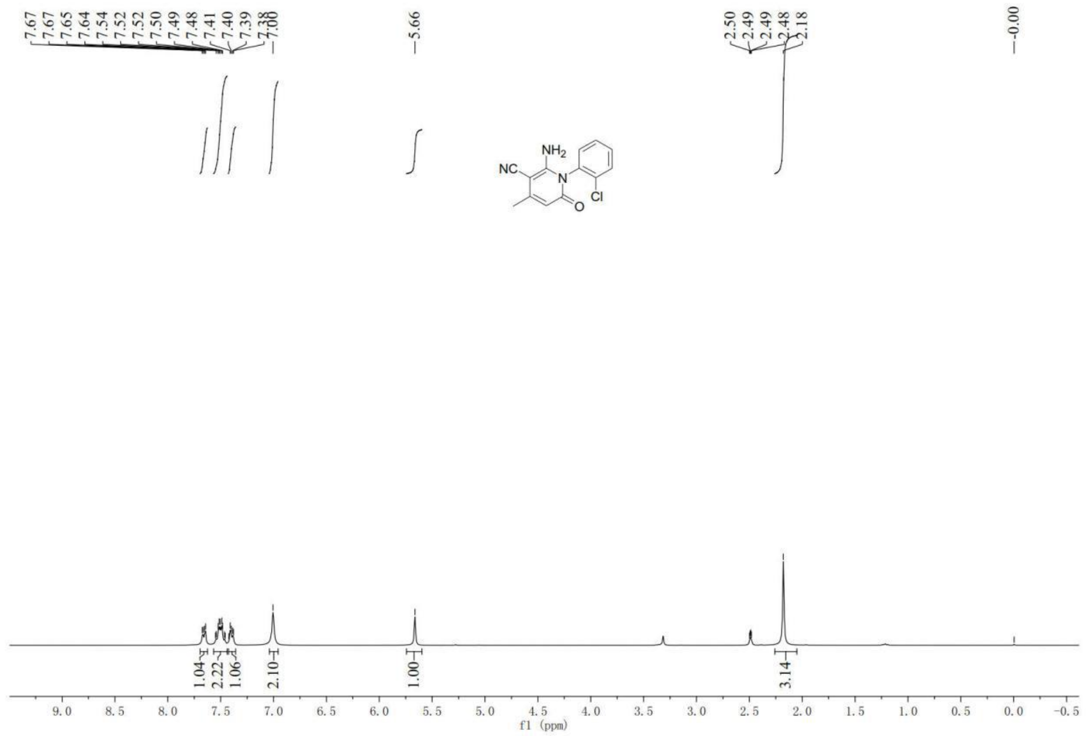
7.67
7.67
7.65
7.64
7.54
7.52
7.52
7.50
7.49
7.48
7.41
7.40
7.39
7.38
7.00
5.66
2.50
2.49
2.49
2.48
2.18
-0.00
NH2
NC
N
Cl
O
1.04
2.22
1.06
2.10
1.00
3.14
f1 (ppm)

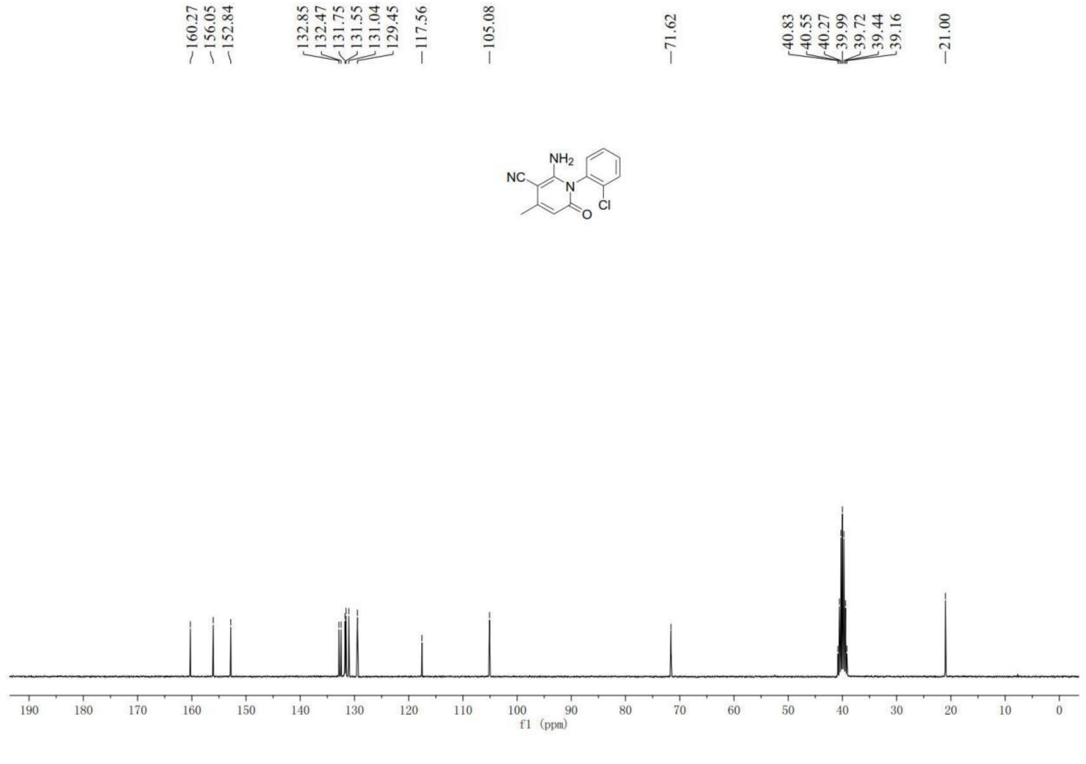
160.27
156.05
152.84
132.85
132.47
131.75
131.55
131.04
129.45
117.56
105.08
71.62
40.83
40.55
40.27
39.99
39.72
39.44
39.16
21.00
NH2
NC
N
Cl
O
f1 (ppm)

Compound **3e**

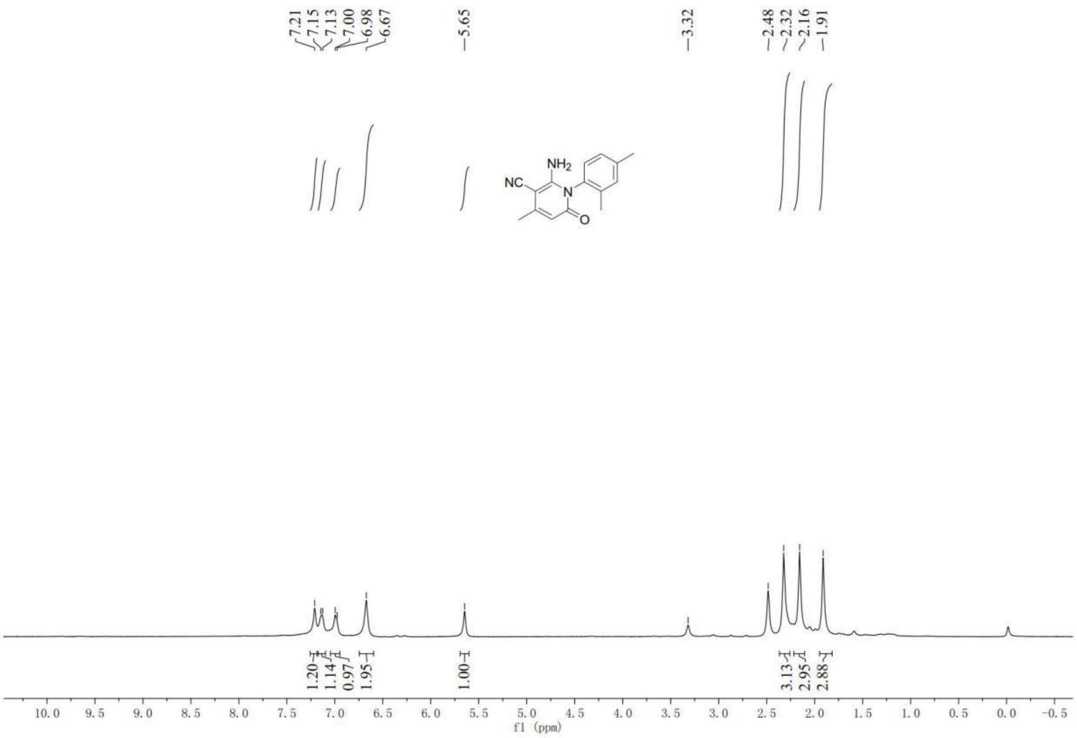

7.21
7.15
7.13
7.00
6.98
6.67
5.65
3.32
2.48
2.32
2.16
1.91
NH2
NC
N
O
1.20
1.14
0.97
1.95
1.00
3.13
2.95
2.88
10.0 9.5 9.0 8.5 8.0 7.5 7.0 6.5 6.0 5.5 5.0 4.5 4.0 3.5 3.0 2.5 2.0 1.5 1.0 0.5 0.0 -0.5
f1 (ppm)

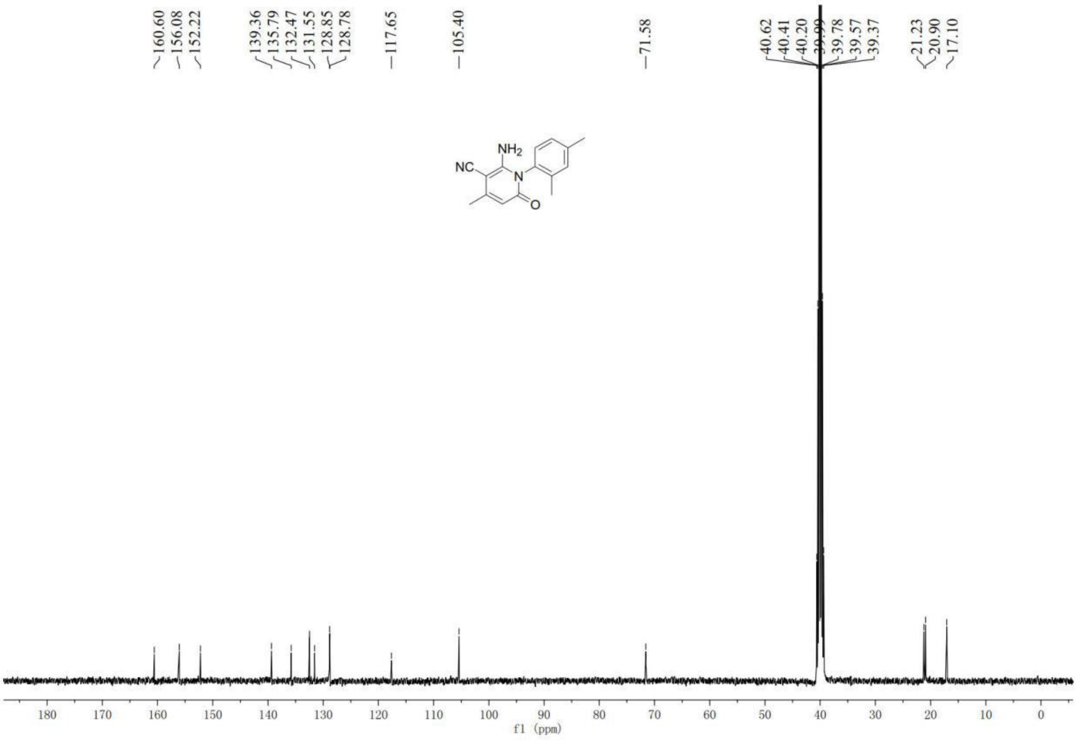

160.60
156.08
152.22
139.36
135.79
132.47
131.55
128.85
128.78
117.65
105.40
71.58
40.62
40.41
40.20
39.78
39.57
39.37
21.23
20.90
17.10
NH2
NC
N
O
180 170 160 150 140 130 120 110 100 90 80 70 60 50 40 30 20 10 0
f1 (ppm)

Compound **3f**

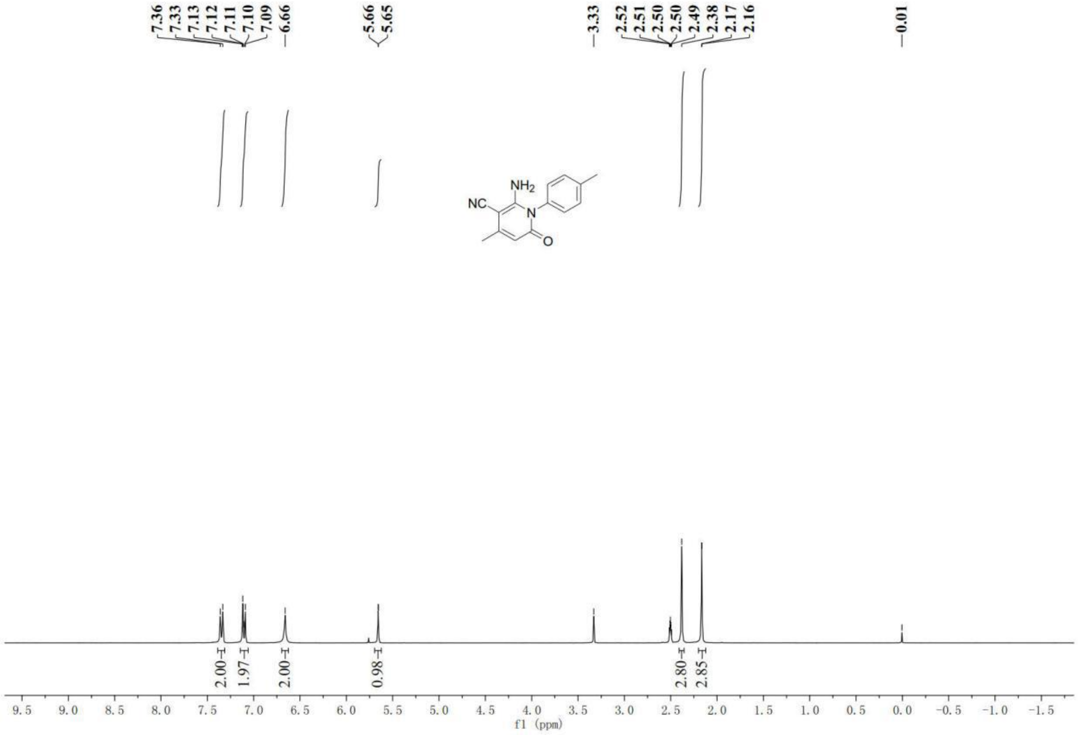

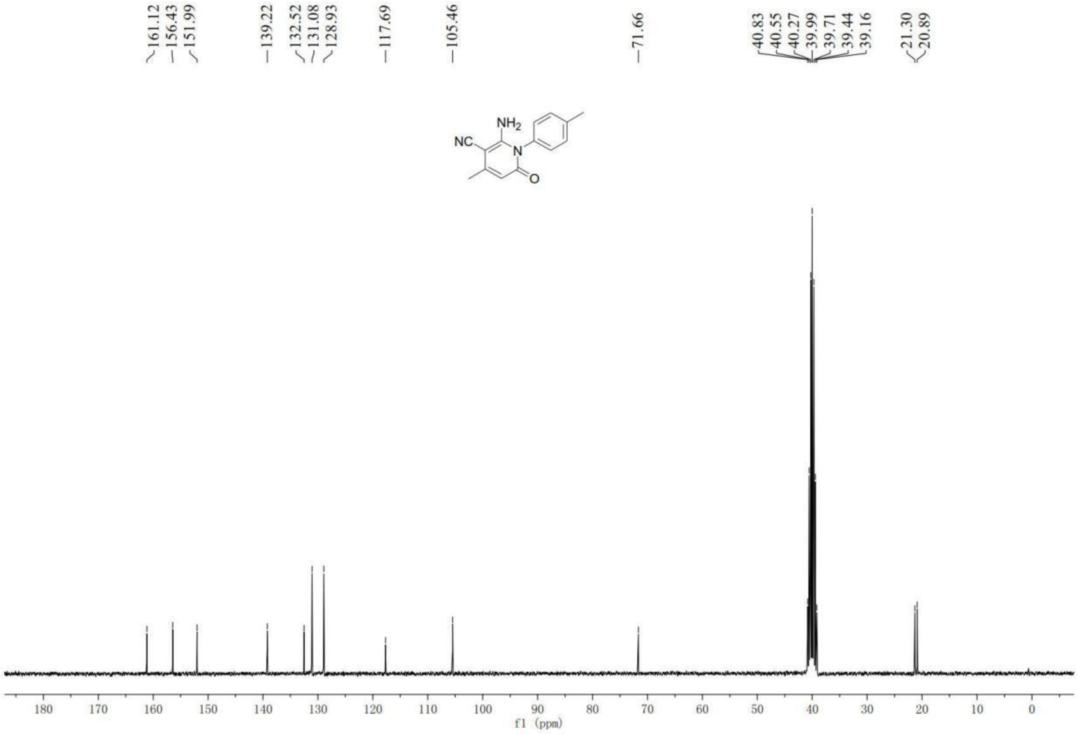

Compound **3g**

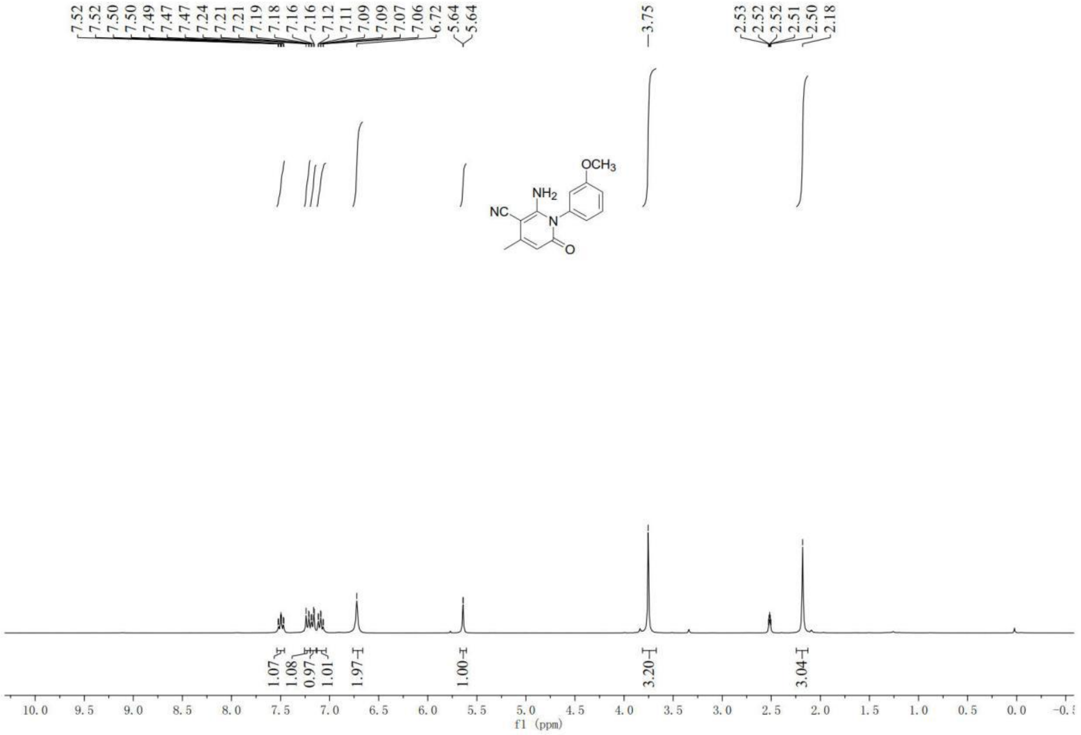

7.52 7.52 7.50 7.50 7.49 7.47 7.47 7.24 7.21 7.21 7.19 7.18 7.16 7.16 7.12 7.11 7.09 7.09 7.07 7.06 6.72 5.64 5.64
3.75
2.53 2.52 2.52 2.51 2.50 2.18
OCH3
NH2
NC
N
O
1.07 1.08 0.97 1.01 1.97 1.00 3.20 3.04
10.0 9.5 9.0 8.5 8.0 7.5 7.0 6.5 6.0 5.5 5.0 4.5 4.0 3.5 3.0 2.5 2.0 1.5 1.0 0.5 0.0
f1 (ppm)

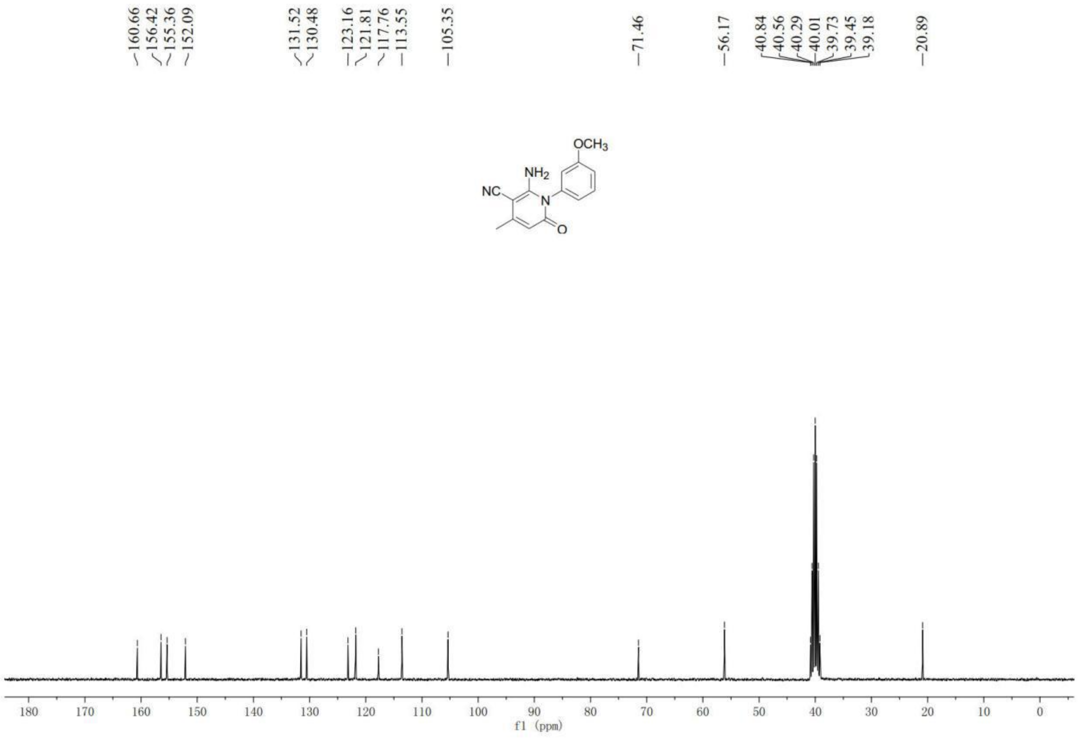

160.66 156.42 155.36 152.09
131.52 130.48
123.16 121.81 117.76 113.55
105.35
71.46
56.17
40.84 40.56 40.29 40.01 39.73 39.45 39.18
20.89
OCH3
NH2
NC
N
O
180 170 160 150 140 130 120 110 100 90 80 70 60 50 40 30 20 10 0
f1 (ppm)

Compound **3h**

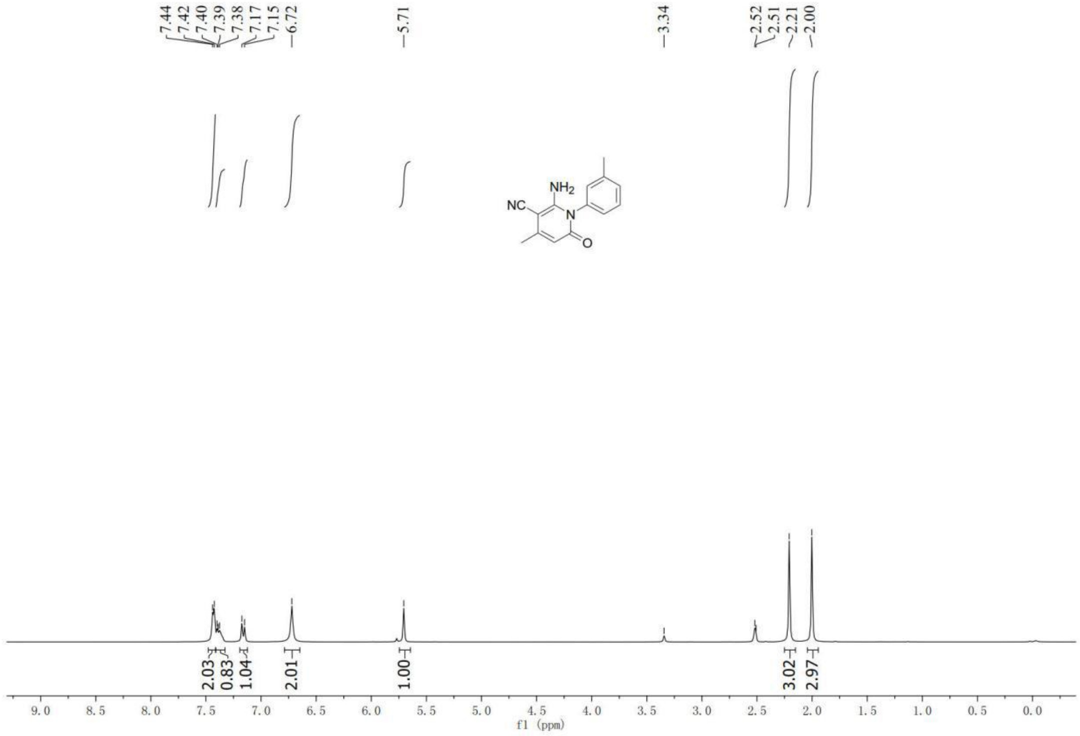
7.44
7.42
7.40
7.39
7.38
7.17
7.15
6.72
5.71
3.34
2.52
2.51
2.21
2.00
NC
NH2
N
O
2.03
0.83
1.04
2.01
1.00
3.02
2.97
9.0 8.5 8.0 7.5 7.0 6.5 6.0 5.5 5.0 4.5 4.0 3.5 3.0 2.5 2.0 1.5 1.0 0.5 0.0
f1 (ppm)

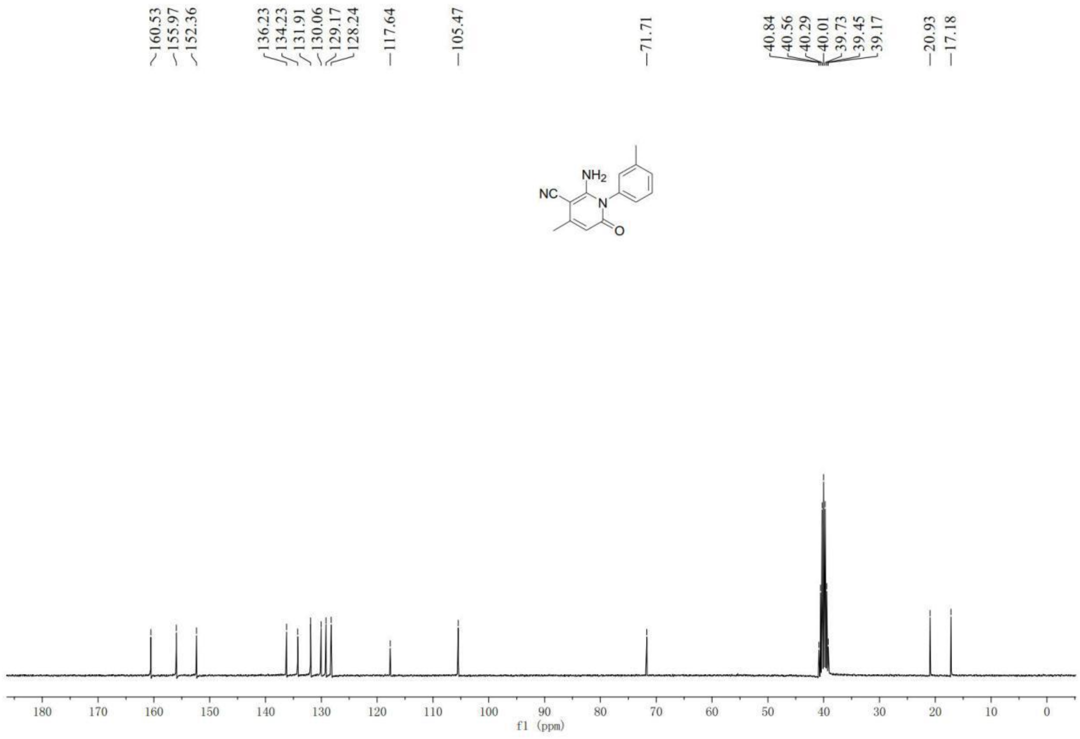
160.53
155.97
152.36
136.23
134.23
131.91
130.06
129.17
128.24
117.64
105.47
71.71
40.84
40.56
40.29
40.01
39.73
39.45
39.17
20.93
17.18
NC
NH2
N
O
180 170 160 150 140 130 120 110 100 90 80 70 60 50 40 30 20 10 0
f1 (ppm)

Compound **4a**

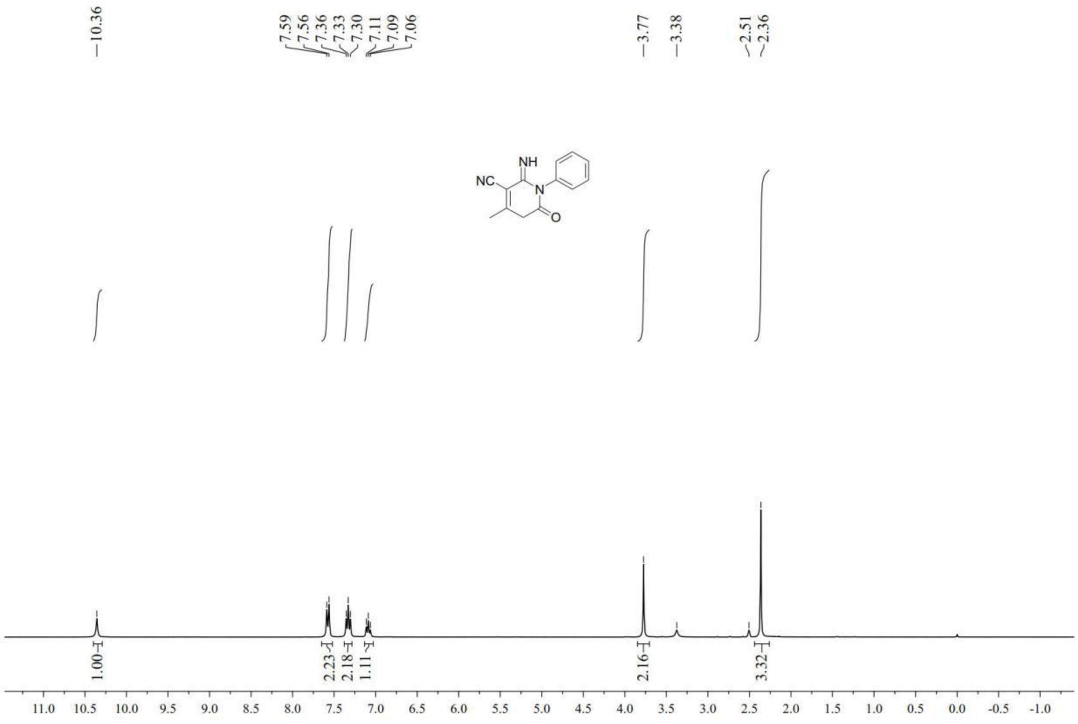
10.36
7.59 7.56 7.36 7.33 7.30 7.11 7.09 7.06
3.77
3.38
2.51 2.36
NH
NC
N
O
1.00
2.23 2.18 1.11
2.16
3.32
11.0 10.5 10.0 9.5 9.0 8.5 8.0 7.5 7.0 6.5 6.0 5.5 5.0 4.5 4.0 3.5 3.0 2.5 2.0 1.5 1.0 0.5 0.0 -0.5 -1.0

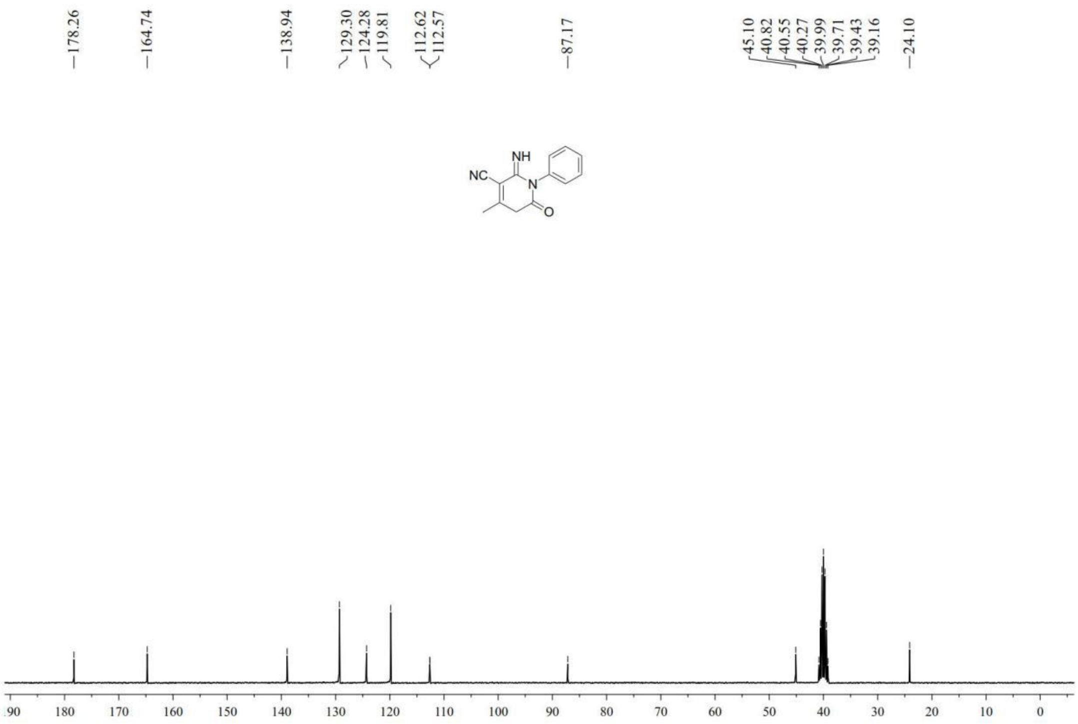
178.26
164.74
138.94
129.30 124.28 119.81
112.62 112.57
87.17
45.10 40.82 40.55 40.27 39.99 39.71 39.43 39.16
24.10
NH
NC
N
O
90 180 170 160 150 140 130 120 110 100 90 80 70 60 50 40 30 20 10 0

Compound **4b**

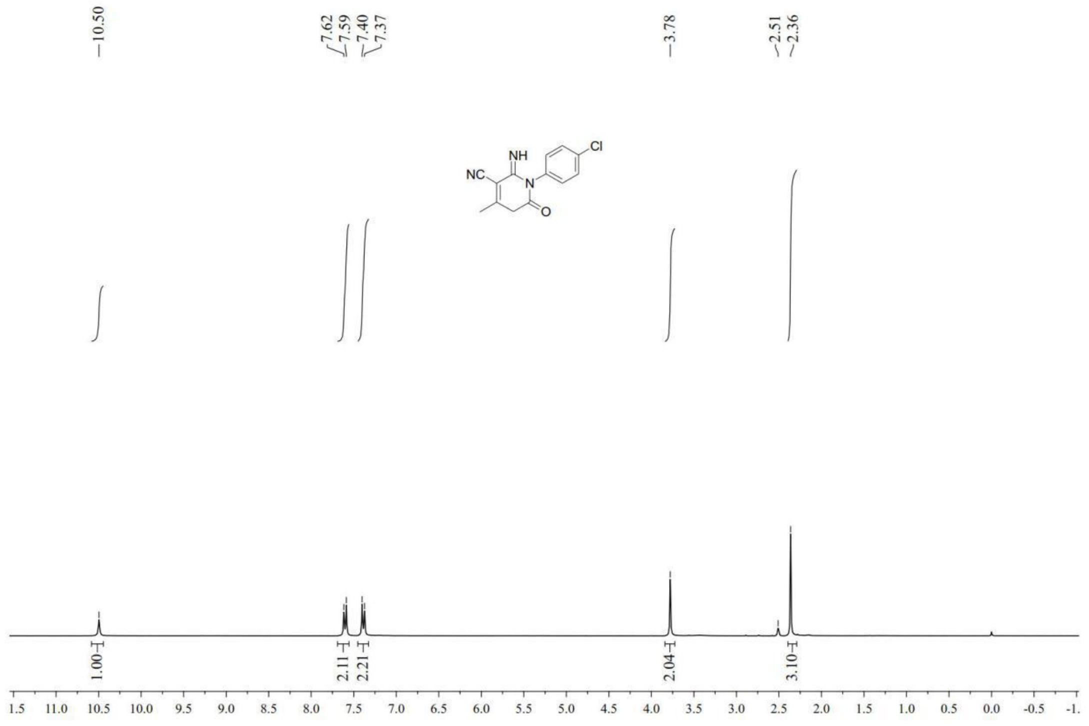
10.50
7.62
7.59
7.40
7.37
3.78
2.51
2.36
1.00
2.11
2.21
2.04
3.10

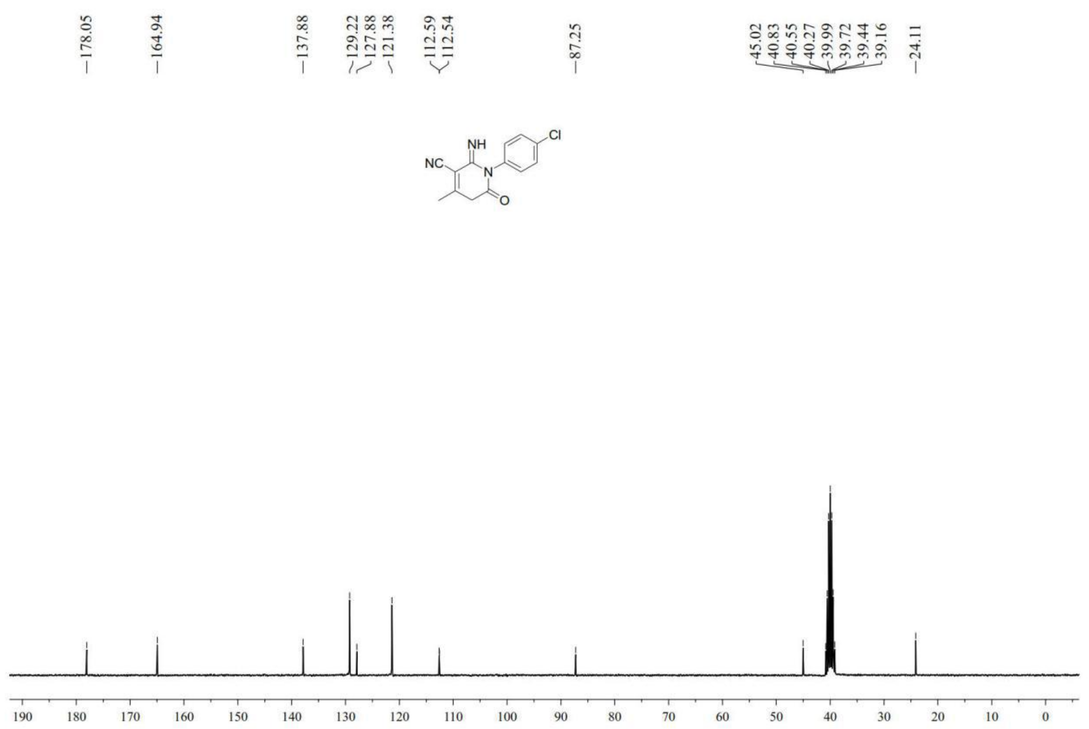
178.05
164.94
137.88
129.22
127.88
121.38
112.59
112.54
87.25
45.02
40.83
40.55
40.27
39.99
39.72
39.44
39.16
24.11

Compound **4c**

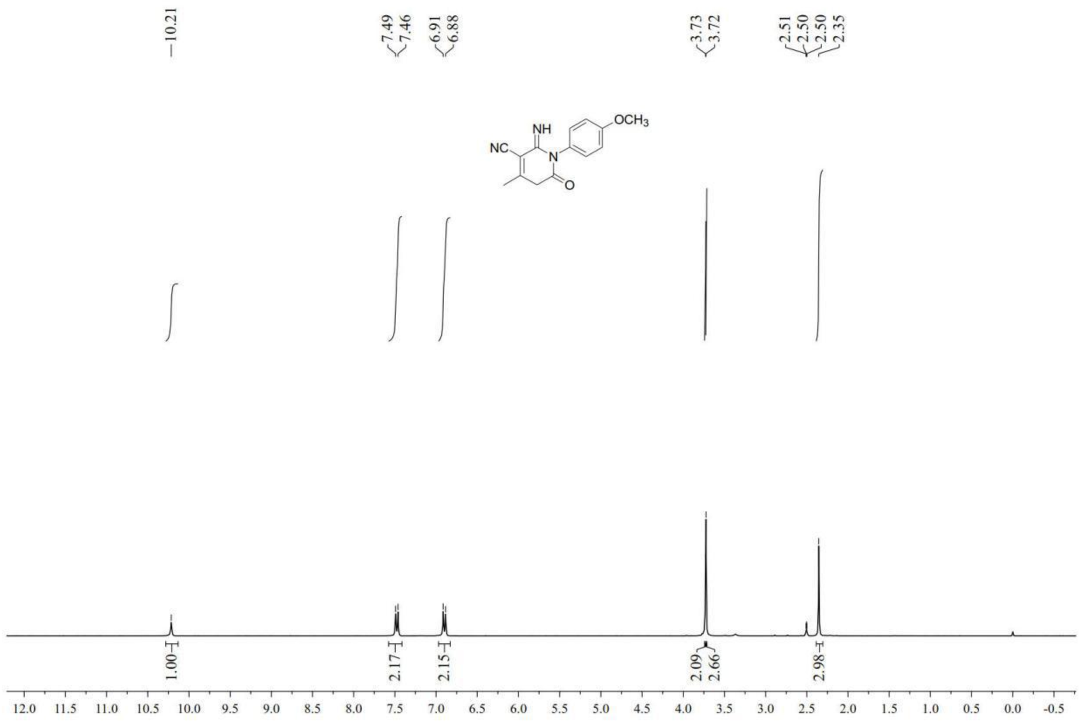
10.21
7.49
7.46
6.91
6.88
3.73
3.72
2.51
2.50
2.50
2.35
NH
OCH3
NC
N
O
1.00
2.17
2.15
2.09
2.66
2.98

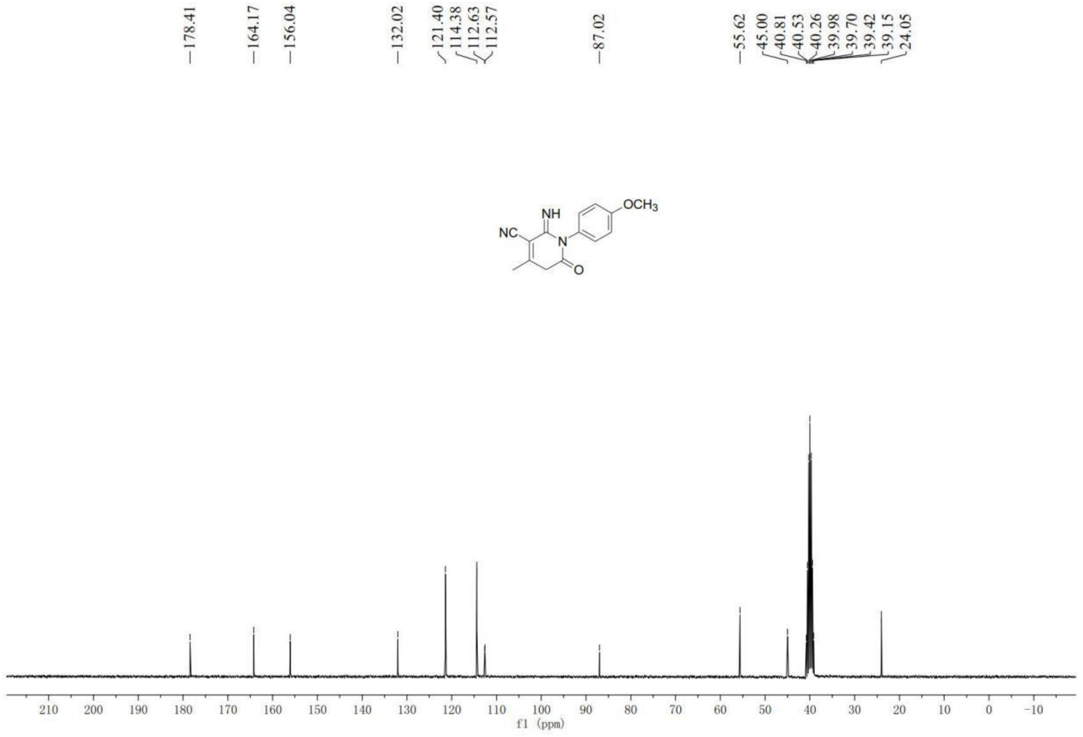
178.41
164.17
156.04
132.02
121.40
114.38
112.63
112.57
87.02
55.62
45.00
40.81
40.53
40.26
39.98
39.70
39.42
39.15
24.05
NH
OCH3
NC
N
O
f1 (ppm)

Compound **4d**

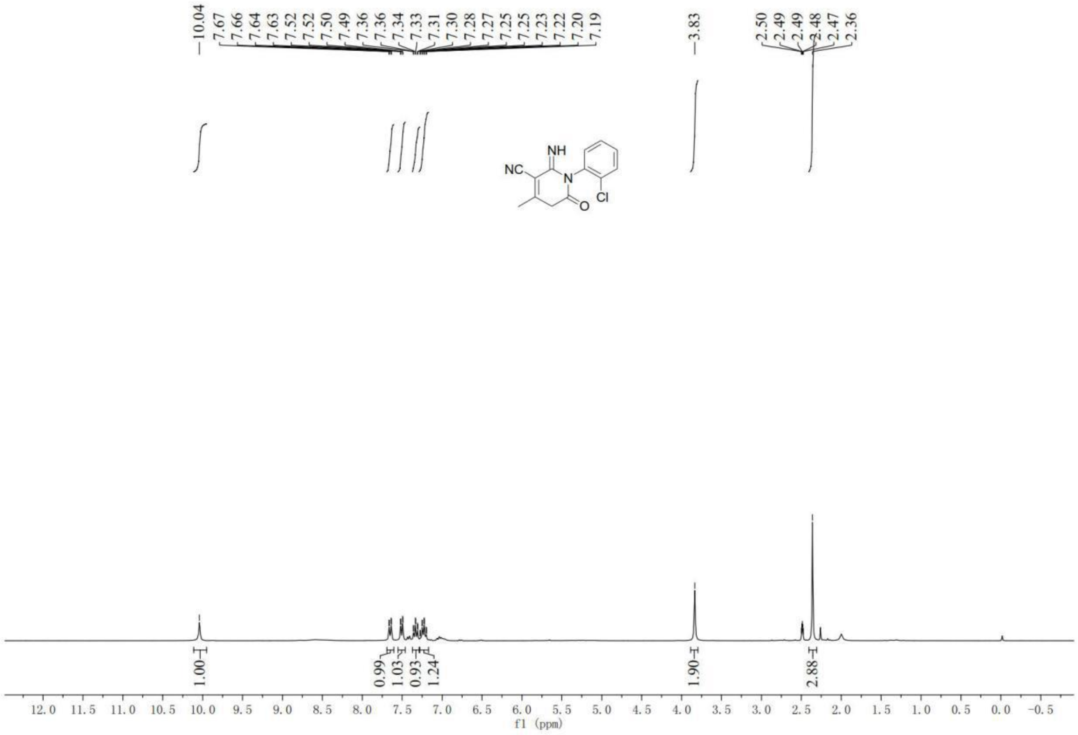

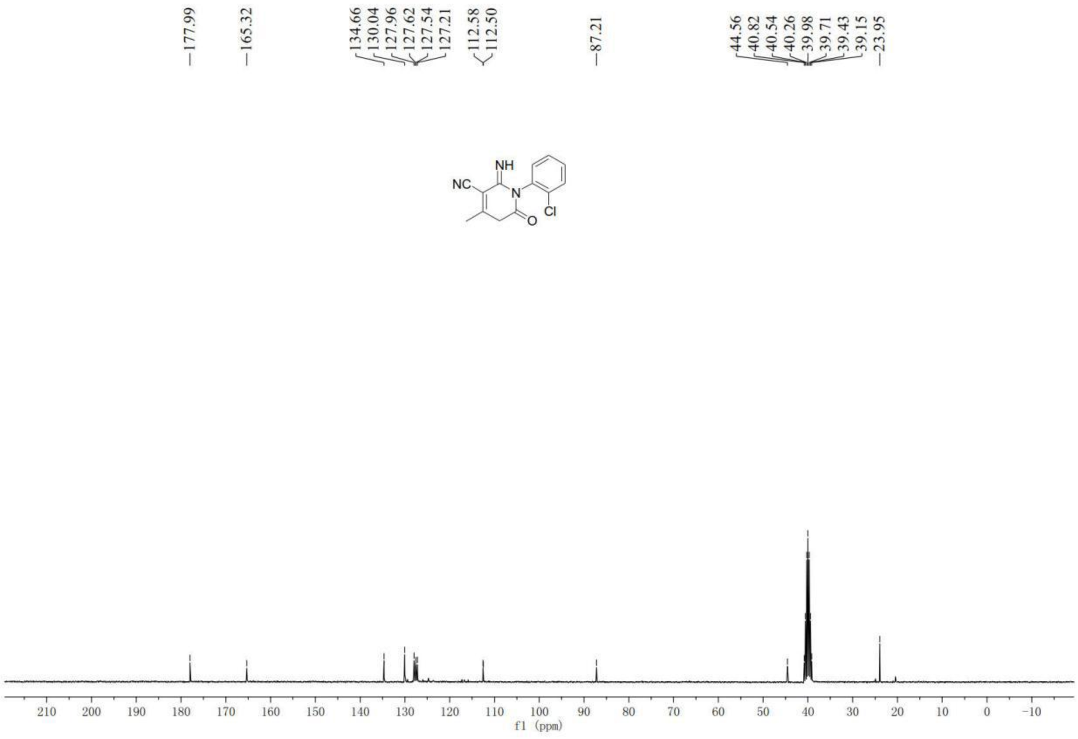

Compound **4e**

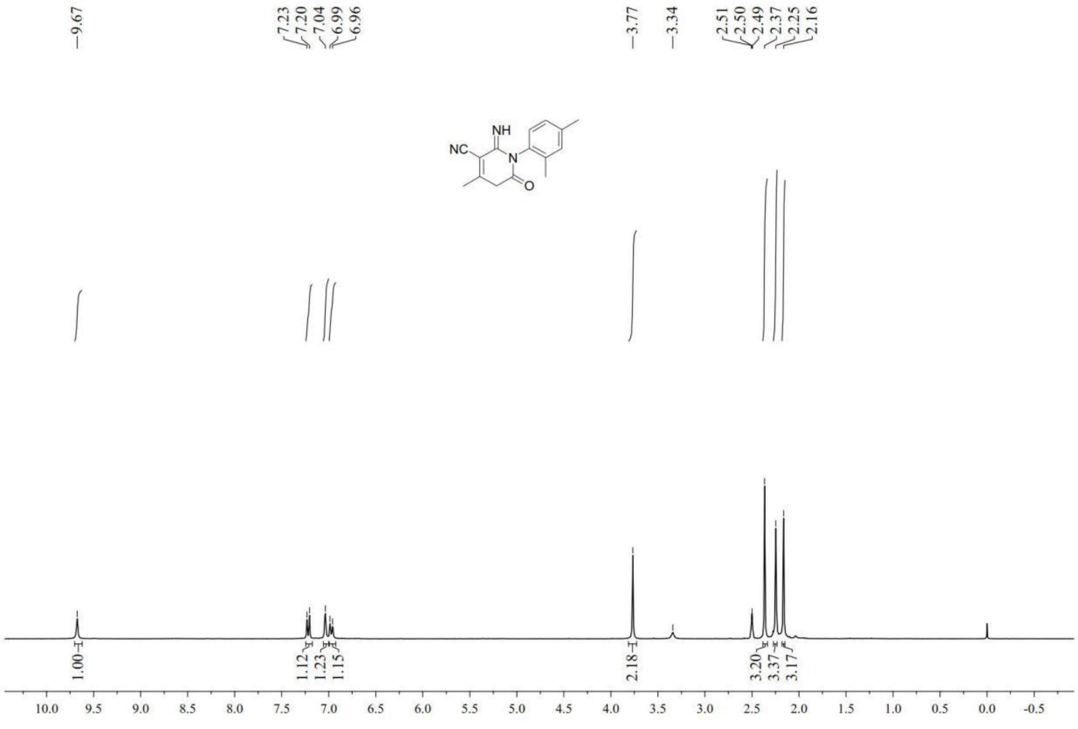

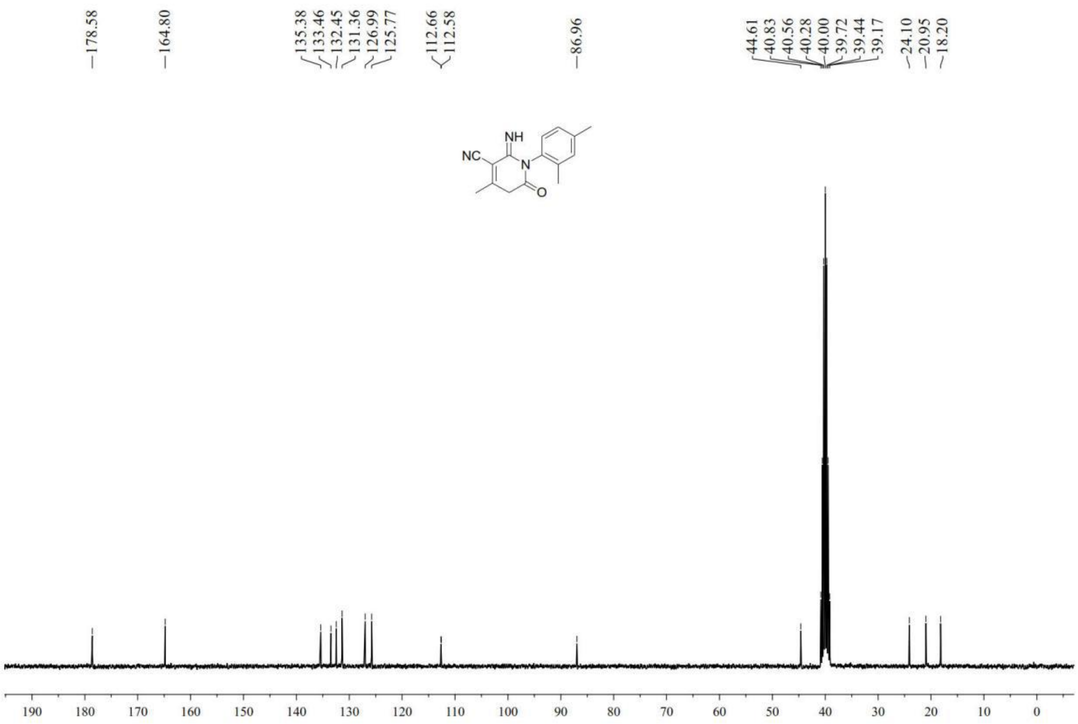

Compound **4f**

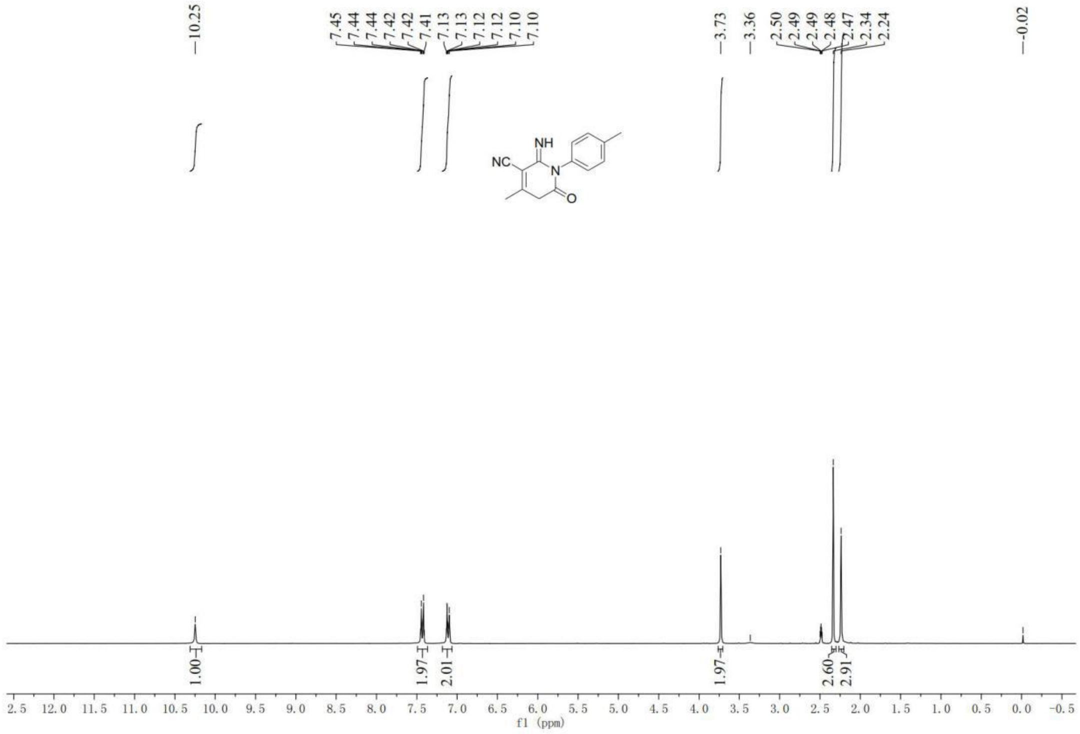

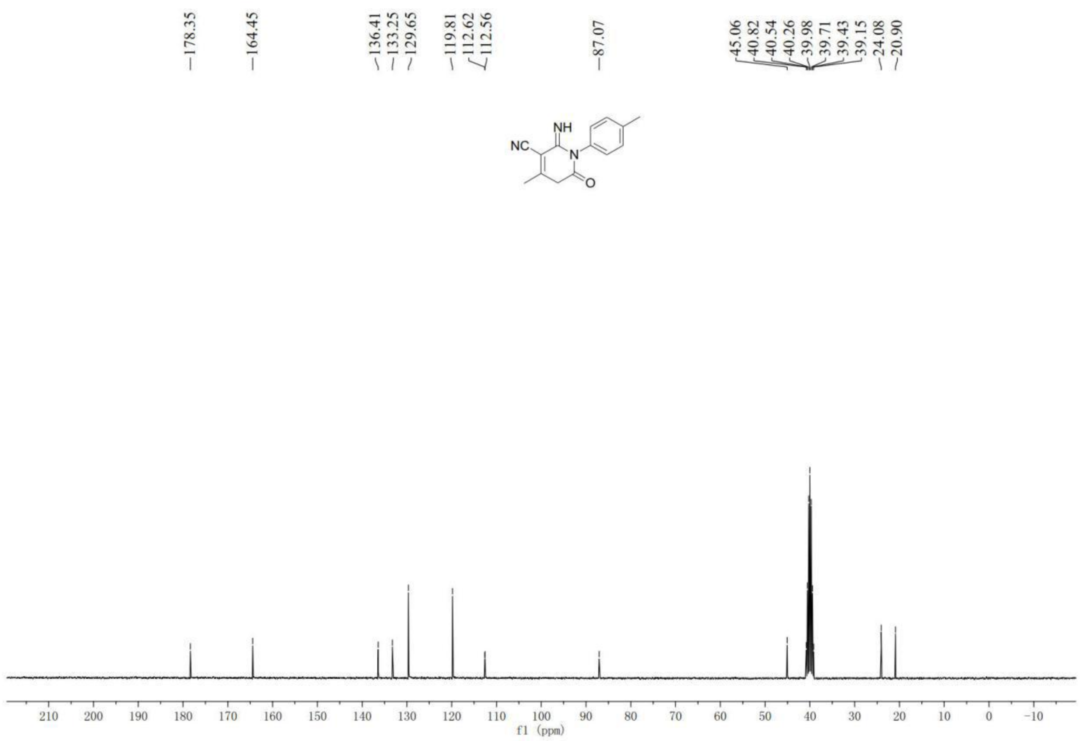

Compound **4g**

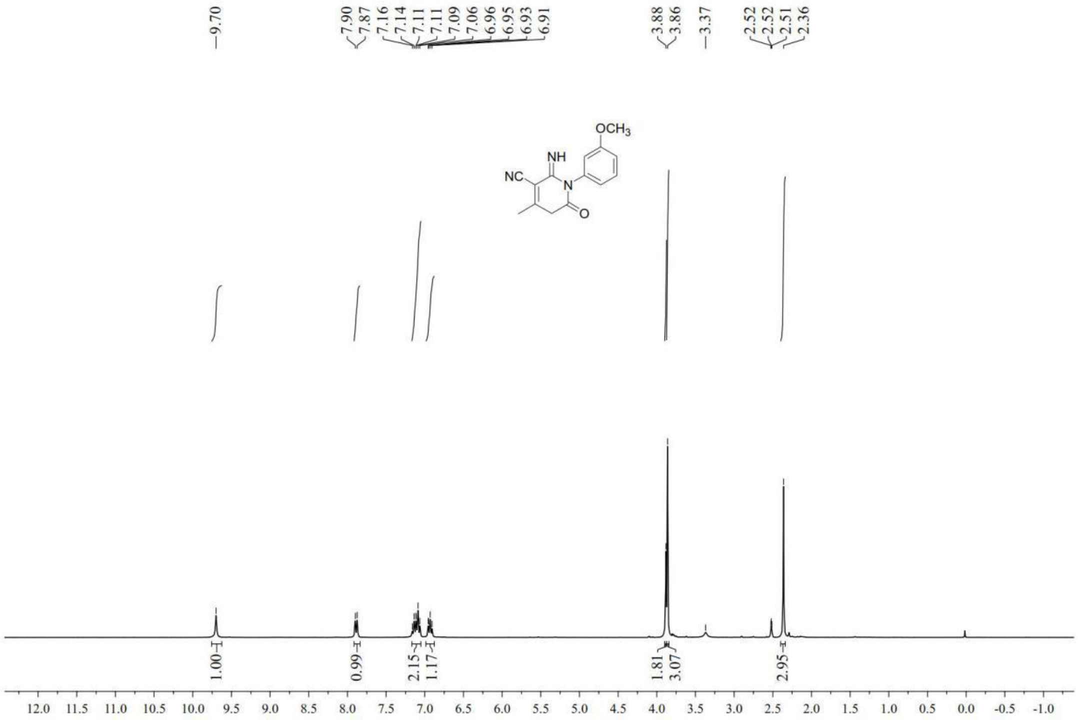
9.70
7.90 7.87 7.16 7.14 7.11 7.11 7.09 7.06 6.96 6.95 6.93 6.91
3.88 3.86
3.37
2.52 2.52 2.51 2.36
OCH3
NH
NC
N
O
1.00
0.99
2.15
1.17
1.81
3.07
2.95
12.0 11.5 11.0 10.5 10.0 9.5 9.0 8.5 8.0 7.5 7.0 6.5 6.0 5.5 5.0 4.5 4.0 3.5 3.0 2.5 2.0 1.5 1.0 0.5 0.0 -0.5 -1.0

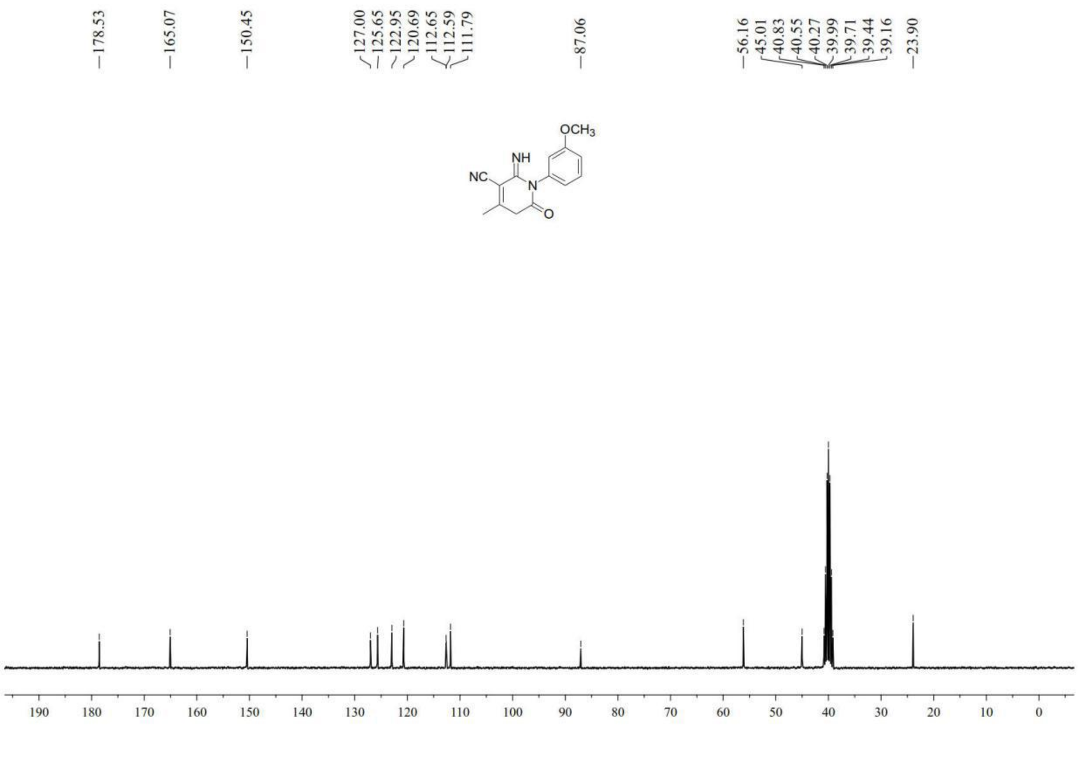
178.53
165.07
150.45
127.00 125.65 122.95 120.69 112.65 112.59 111.79
87.06
56.16 45.01 40.83 40.55 40.27 39.99 39.71 39.44 39.16
23.90
OCH3
NH
NC
N
O
190 180 170 160 150 140 130 120 110 100 90 80 70 60 50 40 30 20 10 0

Compound **4h**

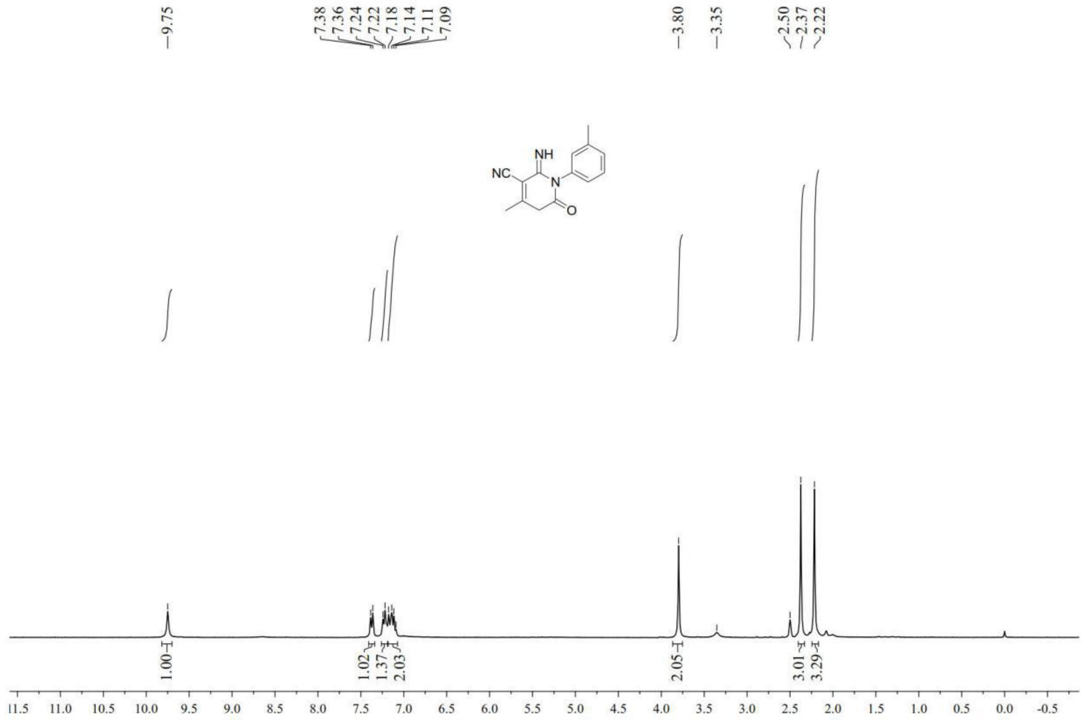
9.75
7.38
7.36
7.24
7.22
7.18
7.14
7.11
7.09
3.80
3.35
2.50
2.37
2.22
NH
NC
N
O
1.00
1.02
1.37
2.03
2.05
3.01
3.29
11.5 11.0 10.5 10.0 9.5 9.0 8.5 8.0 7.5 7.0 6.5 6.0 5.5 5.0 4.5 4.0 3.5 3.0 2.5 2.0 1.5 1.0 0.5 0.0 -0.5

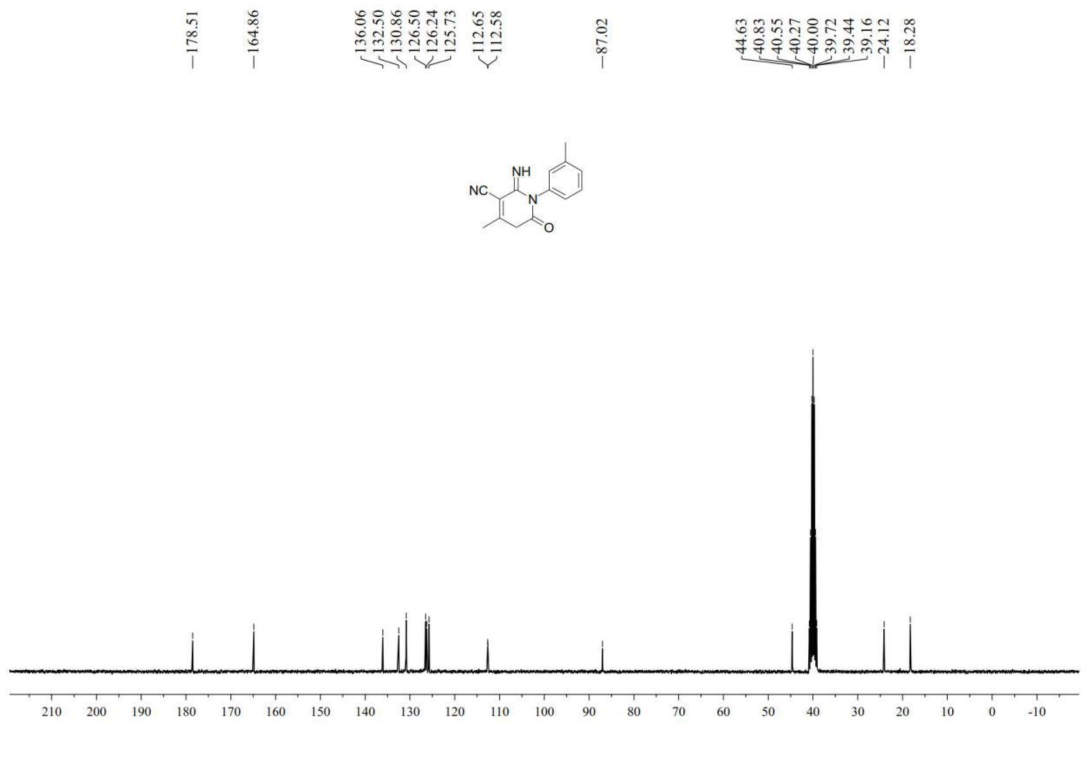
178.51
164.86
136.06
132.50
130.86
126.50
126.24
125.73
112.65
112.58
87.02
44.63
40.83
40.55
40.27
40.00
39.72
39.44
39.16
24.12
18.28
NH
NC
N
O
210 200 190 180 170 160 150 140 130 120 110 100 90 80 70 60 50 40 30 20 10 0 -10
